# O-GlcNAcylated Hsp47 as a predictive biomarker in colorectal cancer: Kaempferol targets OGT-collagen axis for therapeutic intervention

**DOI:** 10.7150/ijbs.116513

**Published:** 2025-08-30

**Authors:** Chishun Zhou, Jing Zheng, Zizheng Li, Yu Li, Xin Jin, Yukai Huang, Yuefang Lin, Xinyue Wen, Yin Wang, Jiarun Lin, Ying Wang, Wei Wang, Zhongqiu Liu, Linlin Lu

**Affiliations:** 1 Guangdong Provincial Key Laboratory of Translational Cancer Research of Chinese Medicines, Joint International Research Laboratory of Translational Cancer Research of Chinese Medicines, State Key Laboratory of Traditional Chinese Medicine Syndrome, State Key Laboratory of Dampness Syndrome of Chinese Medicine, International Institute for Translational Chinese Medicine, School of Pharmaceutical Sciences, Guangzhou University of Chinese Medicine, Guangzhou, 510006, China; Chinese Medicine Guangdong Laboratory (Hengqin Laboratory), Guangdong Hengqin, 519000, China.; 2 Department of Colorectal Surgery, The First Affiliated Hospital of Guangzhou University of Chinese Medicine, Guangdong Clinical Research Academy of Chinese Medicine, Guangzhou 510405, China.; 3 Department of Gastrointestinal Surgery, The First Affiliated Hospital of Guangzhou University of Chinese Medicine, Guangdong Clinical Research Academy of Chinese Medicine, Guangzhou 510006, China.

**Keywords:** Colorectal Cancer, Kaempferol, O-GlcNAc, OGT, Hsp47

## Abstract

Colorectal cancer (CRC) is a highly lethal gastrointestinal malignancy, and its progression is closely related to abnormal protein O-GlcNAcylation modifications, especially during extracellular matrix (ECM) remodeling. Kaempferol is a natural flavonoid with medicinal value that can inhibit CRC progression through various pathways. However, it is unclear whether its mechanism of action involves O-GlcNAc-driven metabolic reprogramming. This study confirmed that kaempferol can significantly inhibit CRC growth both *in vitro* and *in vivo* and effectively reduce the overall protein O-GlcNAcylation levels. Mechanistic studies indicate that kaempferol reduces the levels of substrate uridine diphosphate N-acetylglucosamine (UDP-GlcNAc) and downregulates the expression of O-GlcNAc transferase (OGT), thereby decreasing the O-GlcNAcylation levels of proteins. This leads to a reduction in the O-GlcNAc modification of downstream heat shock protein 47 (Hsp47), which in turn affects the expression and intracellular localization of Hsp47, ultimately inhibiting the maturation and secretion of type I collagen, thereby blocking CRC progression. This study reveals a new mechanism by which kaempferol inhibits CRC by targeting the O-GlcNAcylation pathway. The study results suggest that O-GlcNAc-modified Hsp47 could serve as a potential therapeutic target for CRC and propose a treatment strategy guided by flavonoid biomarkers based on the inhibition of the OGT-collagen axis.

## Introduction

Colorectal cancer (CRC) is the leading malignancy of gastrointestinal cancers (GI) and has the highest incidence and mortality rates 1. Overwhelming evidence has demonstrated that progression, metastasis, recurrence, and chemotherapy sensitivity in CRC patients are closely associated with glycosylation, one of the crucial posttranslational modifications (PTMs) frequently observed in approximately 30%-40% of CRC tissues 2-3. By adding carbohydrate molecules to functional proteins, glycosylation significantly influences CRC by altering the function, stability, and intracellular localization of proteins 4. Depending on the type of glycosylation bond formed, glycosylation can be categorized into four types: N-glycosylation, O-glycosylation, C-mannosylation, and glycosylphosphatidylinositol (GPI) anchoring 5. Among these, O-GlcNAcylation, which attaches GlcNAc to serine or threonine residues on proteins, predominantly contributes to CRC progression by dynamically regulating nearly 80% of key CRC-associated proteins involved in metabolic reprogramming, signal transduction, and extracellular matrix (ECM) remodeling 6. The ECM, a determinant in the tumor microenvironment, controls CRC progression and metastasis by providing structural support and signaling cues and influencing cell behavior. It is estimated to contribute up to 40%-50% of the mechanisms driving CRC progression and metastasis 4. Collagen, a major component of the ECM, relies on O-GlcNAcylation for proper assembly and deposition 7, whereas numerous studies have indicated that collagen itself frequently undergoes N-glycosylation. Therefore, whether collagen-associated proteins in the ECM can inhibit CRC progression and metastasis through O-GlcNAcylation remains largely unknown.

Heat shock protein 47 (Hsp47), encoded by the SERPINH1 gene, is a collagen-specific molecular chaperone that is essential for the proper folding and assembly of collagen in the endoplasmic reticulum. Hsp47 facilitates the maturation and secretion of procollagen, thereby maintaining ECM integrity and promoting tissue structural stability, which are critical factors for cancer progression and metastasis 8. In CRC, Hsp47 expression is significantly upregulated, with recent studies suggesting that it could influence approximately 30-40% of the ECM remodeling process 9. This, in turn, supports tumor survival, chemoresistance, and invasiveness through pathways such as the AKT 10, CCL2 11, and Wnt/β-catenin signaling pathways 12, making Hsp47 a potential prognostic biomarker and therapeutic target for CRC patients. Additionally, Hsp47 undergoes posttranslational modifications, including phosphorylation, which further influence its chaperone function and stability within the cell, potentially affecting tumor progression in CRC. Moreover, previous studies have demonstrated that Hsp47 mutations can determine the glycosylation and/or hydroxylation of type I procollagen 13. However, whether the Hsp47 protein itself undergoes glycosylation and the effect of its modification on CRC progression and metastasis have not yet been reported.

Kaempferol, a common but enriched polyphenol compound found in the medicinal-food homologous herb *Kaempferia galanga* L., has emerged as the most promising plant-derived lead compound for the development of anti-CRC agents due to its high efficacy in clinical prescriptions against CRC. Previous studies have shown that kaempferol can influence the structure, function, and remodeling of the ECM through multiple mechanisms: (i) Kaempferol downregulates the MMP 14 and interacts with ASIC1a 15 and Krt-14 16 to increase collagen crosslinking and accumulation, which attenuates tumor cell migration 17; (ii) kaempferol regulates cancer metabolic reprogramming by inhibiting glycolysis through targeting HK2 and PKM2, as well as reducing fatty acid synthesis; (iii) kaempferol contributes to ECM remodeling and collagen synthesis by modulating signaling pathways such as the NF-κB, TGF-beta, Nrf2, PI3K/AKT, and ER stress pathways 18-19. However, whether kaempferol can directly affect collagen formation and inhibit CRC metastasis by regulating the glycosylation of collagen-related proteins remains unclear.

In this study, we elucidated the mechanism by which kaempferol inhibits CRC progression and metastasis by targeting the O-GlcNAcylation pathway. On one hand, kaempferol reduces the production of UDP-GlcNAc, and on the other hand, it directly targets and inhibits the expression and activity of OGT, thereby reducing the O-GlcNAcylation level of the downstream protein Hsp47. This ultimately inhibits the formation of type I collagen, alleviating CRC progression and metastasis. (i) Tissue microarray, immunofluorescence, and immunohistochemical analyses show that O-GlcNAcylation is significantly elevated in clinical CRC tissues, and its level is closely related to CRC progression and poor prognosis. Kaempferol treatment effectively inhibits the level of O-GlcNAcylation in tumor tissues. (ii) Molecular dynamics simulations, SIP/CETSA, and key amino acid site mutation experiments of OGT confirmed that kaempferol directly binds to the His-901 and Asp-925 residues of OGT, thereby inhibiting OGT's enzymatic activity and its expression. (iii) Bioinformatics analysis, immunoprecipitation (IP), and Click-iT O-GlcNAc enzyme-linked enrichment assays indicate that kaempferol significantly reduces the O-GlcNAcylation modification at the Ser76 site of the Hsp47 protein. The reduction in this modification level disrupts the Hsp47-mediated maturation and intracellular transport of type I collagen, ultimately hindering the progression and metastasis of CRC. This study not only reveals for the first time that collagen-related molecular chaperone proteins in the ECM can undergo O-GlcNAcylation modifications, but also provides a theoretical basis for biomarker-guided CRC intervention strategies targeting the OGT-Hsp47-collagen axis.

## Materials and Methods

### Cells

Human colorectal cancer cells HCT116 (C1125), Caco2 (C1115), HT29 (C1007), SW480 (C1128), and human normal colon epithelial cells HCoEpiC (C1228) and NCM460 (C1227) were purchased from Shanghai Meiwan Biotechnology Co., Ltd.; human colorectal adenocarcinoma cells DLD-1 (CL-0074) and RKO (CL-0196) were purchased from Wuhan Procell Biotechnology Co., Ltd.; mouse colorectal cancer cells MC38 (C2088) were purchased from Shanghai Yingwan Biotechnology Co., Ltd. All the above cells have been authenticated as the corresponding cell lines by STR DNA profiling. According to the manufacturers' instructions, cells were cultured in DMEM (Gibco) or RPMI 1640 (Gibco) medium supplemented with 1% penicillin‒streptomycin and 10% fetal bovine serum (FBS, Gibco), and incubated in a 37°C incubator with 5% CO2.

### Animals

Male APC^Min/+^ and male C57BL/6 mice, aged 5-6 weeks and of SPF grade, were purchased from the Guangdong Provincial Medical Laboratory Animal Center. They were housed in the SPF-grade animal facility of the International Research Institute of Traditional Chinese Medicine Transformational Medicine at Guangzhou University of Chinese Medicine, with the facility operating under license number: SYXK(Yue) 2024-0144. The animals had free access to water and food and were maintained in an environment with a temperature of 22 ± 2°C, a relative humidity of 60 ± 10%, and a 12-hour light/dark cycle. All animal experiments were approved by the Care and Use Committee of the International Research Institute of Traditional Chinese Medicine Transformational Medicine at Guangzhou University of Chinese Medicine.

### Patient samples

CRC samples were collected from the First Affiliated Hospital of Guangzhou University of Chinese Medicine. All patients provided written informed consent, in accordance with the principles of the Declaration of Helsinki. The research involving human tissues was approved by the Ethics Committee of Guangzhou University of Chinese Medicine (Ethics number: K-2023-010).

### Antibodies

Anti-O-GlcNAc (PTM-951RM), Anti-benzoyllysine (PTM-762), Anti-L-Lactyl Lysine (PTM-1401RM), Anti-crotonyllysine (PTM-501), Anti-3-nitrotyrosine (PTM-752), Anti-succinyllysine (PTM-401), Anti-acetyllysine (PTM-101), Anti-ubiquitin (PTM-1106RM), Anti-phosphotyrosine (PTM-702RM), Anti-sumo1/2/3 (PTM-1109), Anti-malonyllysine (PTM-901), Anti-OGT (PTM-5497), Anti-HK2 (PTM-5371) were purchased from Jingjie PTM BioLab (Hangzhou) Co. Ltd.; Hsp47 (60448S), α-Tubulin (2144S) were purchased from Cell Signaling Technology; Anti-ARL8A (ab270979), Anti-Vinculin (ab129002), Rabbit Anti-Mouse IgG H&L (HRP) (ab6728), Goat Anti-Rabbit IgG H&L (HRP) (ab6721), Anti-Lamin B1 (ab229025) were purchased from Abcam; O-GlcNAc transferase (sc-74547), NNMT (sc-376048), E-cadherin (sc-8426), N-cadherin (sc-8424), Calnexin (sc-23954), Sox2 (sc-365823), P53 (sc-126), OCT-4 (sc-5279), MMP9 (sc-21733) were purchased from Santa Cruz Biotechnology; Anti-MGEA5/OGA (14711-1-AP), CoraLite^®^594-Phalloidin (PF00003), Alexa Fluor 555 conjugated Donkey Anti-Rabbit IgG (H+L) (A0453), Alexa Fluor 647 conjugated Goat Anti-Mouse IgG (H+L) (A0473), Alexa Fluor 488 conjugated Goat Anti-Rabbit IgG (H+L) (A0423), CoraLite^®^555-conjugated GM130 (CL555-11308), CoraLite^®^ Plus 555-conjugated calreticulin (CL555-27298), DYKDDDDK tag Recombinant antibody (Binds to FLAG^®^ tag epitope)(80010-1-RR) were purchased from Proteintech Group; Human Pro Collagen I alpha 1 Antibody (MAB6220) was purchased from R&D Systems.

### Western blotting and immunoprecipitation

Cells were harvested and lysed using RIPA buffer (Thermo Fisher Scientific, USA) after washing with PBS. The supernatant was collected via centrifugation, and protein concentrations were determined using a BCA assay kit (Beijing Dingguo Changsheng Biotechnology Co., Ltd., China). An 8%-12% SDS‒PAGE gel was prepared for electrophoretic separation of the proteins, which were then transferred onto a polyvinylidene fluoride (PVDF) membrane (Merck, Germany). After blocking with 5% skim milk, the membrane was incubated with the primary antibody at a dilution of 1:1000 and the secondary antibody at a dilution of 1:5000. Imaging was carried out using an ECL reagent with the Tanon 5200 chemiluminescence imaging system (Tanon, Shanghai, China). For immunoprecipitation, the protein lysate was incubated with antibody-magnetic bead complexes, washed with lysis buffer, and then boiled for subsequent analysis by Western blotting.

### Wounding

Confluent cells in a 6-well plate were treated with 0.4 g/mL mitomycin (Sigma‒Aldrich) for 30 minutes to inhibit cell proliferation. A scratch was then made on the cell monolayer using a 10 μL pipette tip, and the cells were washed with PBS to remove debris. The cells were subsequently cultured in DMEM containing gentamicin (MeilunBio, MB2171) and 1% fetal bovine serum (FBS). Photographs were taken at 0, 12, 24, and 48 hours to observe and analyze cell migration ability.

### Transwell

Transwell culture plates were purchased from Corning (New York, USA). Cells were starved and cultured overnight in a chamber with a diameter of 24 mm and a pore size of 8 μm. In the lower chamber, 500 μL of complete culture medium was added and incubated for 48 hours. Migrated cells were fixed in 4% formaldehyde for 15 minutes, stained with 0.1% crystal violet for 10 minutes, and then photographed under a microscope.

### Colony formation

Cells were diluted to a concentration of 100 cells/mL and plated in a 6-well plate. They were then cultured for 14 days, with the medium replaced every 3 days. After the culture period, the old medium was discarded, and the cells were washed with PBS. The cells were fixed for 15 minutes and stained with crystal violet (Beyotime Biotechnology, C0121) for 15 minutes, followed by washing off the excess stain and air-drying. Finally, photographs were taken to record the number of cell colonies.

### Hematoxylin-Eosin (HE) staining

Colorectal tissue was fixed in 4% paraformaldehyde, rinsed overnight, and then cleaned with PBS. It was subsequently subjected to gradient dehydration with ethanol and xylene before being embedded in both soft and hard paraffin wax. The paraffin-embedded tissue block was precooled, and 4 μm-thick sections were cut, spread out, and fixed onto slides. HE staining was then performed, which included deparaffinization, rehydration, staining of cell nuclei with hematoxylin, and staining of the cytoplasm with eosin. Finally, the slides were cleared, mounted, and photographed for documentation.

### Immunohistochemistry (IHC)

After being fixed in formalin, the paraffin-embedded tissue sections were baked at 60°C for 2 hours, dewaxed with xylene, and rehydrated with a gradient of ethanol. Sodium citrate was used for heat-induced epitope retrieval at 95°C for 20 minutes, followed by natural cooling and PBS washing. 3% H_2_O_2_ was used to block endogenous peroxidase for 10 minutes, followed by three washes with PBS; 3% BSA was used to block nonspecific binding at room temperature for 30 minutes. The primary antibody was added and incubated overnight at 4 °C, washed with PBS, then the HRP-conjugated secondary antibody was added and incubated at room temperature for 30 minutes. DAB chromogenic reaction, hematoxylin counterstaining, ethanol dehydration, xylene clearing, and neutral gum mounting were then performed. ImageJ was used to evaluate staining intensity by measuring the integrated optical density (IOD) value and the area value of each image, and then calculating the mean density value, i.e., mean density = IOD/area. This value reflects the expression of the target protein per unit area. Finally, the average mean density of 5 random regions for each sample was taken as the value for that sample. In addition, IHC tissue chips were from Xi'an Zhongke Guanghua Bioaitech Co., Ltd.

### 5-Ethynyl-2'-deoxyuridine (EdU)

Cells were seeded in 96-well plates (5×10³ cells/well) and cultured for 24 hours. EdU (10 μM) was added for 2 h incubation. After fixation with 4% PFA (15 min, RT) and permeabilization with 0.5% Triton X-100 (20 min, RT), cells were incubated with Click-iT^®^ reaction cocktail (30 min, dark). Nuclei were counterstained with DAPI (1 μg/mL, 10 min). Fluorescence images were acquired and analyzed to determine EdU-positive cell percentages.

### Quantitative real-time polymerase chain reaction (qRT-PCR)

Total RNA was extracted using TRIzol reagent (Hunan Acres (AG) Biotechnology Co., Ltd.), and reverse transcription was performed with a reverse transcription kit. Quantitative real-time PCR (qRT-PCR) experiments were conducted using the QuantStudio^®^ detection system (Thermo Fisher Scientific, USA) and the SYBR^®^ Green Pro Taq HS premixed qPCR reagent kit. The primer sequences used are shown in Table 1.

### LC/MS analysis

Under light protection and nitrogen, 3.0 mL of trimethylsilyldiazomethane was added to 9.75 mL of a methanol/water mixture (volume ratio 3:25) and mixed for later use. After trypsin digestion of the cells, the cells were washed with PBS, counted, and sonicated in a methanol/water mixture (volume ratio 12:1). The supernatant was collected after centrifugation. Then, mix 100 μL of the supernatant, 10 μL of the internal standard probenecid (concentration 10 mM/L), and 100 μL of the derivatization reagent, and react for 30 minutes. Next, evaporate to dryness in a TurboVap evaporator, resuspend in 50% acetonitrile, and centrifuge. Inject 10 μL of the sample into the LC/MS equipment and analyze the content of UDP-GlcNAc using a mobile phase mixture of ammonium acetate and acetonitrile 20. LC‒MS analysis was performed using an Agilent 1200 series liquid chromatograph coupled to an Agilent 6120 quadrupole mass spectrometer. The chromatographic separation was achieved on a C18 column (5 μm, 4.6 × 250 mm) with a mobile phase consisting of 0.1% formic acid in water (A) and 0.1% formic acid in acetonitrile (B). The gradient elution program was set as follows: 0-5 min, 95% A; 5-15 min, 5% A; 15-20 min, 5% A. The flow rate was 1.0 mL/min, and the column temperature was maintained at 30°C. The mass spectrometer was operated in positive ion mode.

### Cell proliferation

Cells were cultured in a 96-well plate and treated with various concentrations of kaempferol for 24, 48, and 72 hours. They were then fixed at 4°C for 4 hours. After rinsing with deionized water, the cells were stained with Sulforhodamine B (SRB), decolorized with 1% acetic acid, air-dried at room temperature, and the optical density (OD) was measured at a wavelength of 540 nm.

### Glucose uptake assay

Cells are seeded in a 96-well plate and treated with kaempferol for 48 hours. Following treatment, the cells are rinsed with prewarmed glucose-free culture medium and incubated for 15 minutes in a 37 °C, 5% CO2 incubator. After removing the supernatant, the cells are incubated with the probe solution from the Glucose Uptake Assay Kit - Blue (DOJINDO, UP01) for 15 minutes. They are then rinsed with precooled Wash Buffer (WI) solution and allowed to stand for 5 minutes. The fluorescence intensity is measured using a confocal microscope to assess the amount of glucose uptake.

### Cycloheximide (CHX) assay

After removing the old cell culture medium and washing the cells, complete culture medium containing CHX (100 μM), kaempferol (100 μM), MG132 (10 μM), and chloroquine (10 μM) is added. Cells are harvested at 0, 24, 48, and 72 hours to extract cellular proteins, which are then analyzed by Western blotting.

### SIP (Solvent-Induced Protein Precipitation) and CETSA (Cellular Thermal Shift Assay)

Adjust the protein solution to a concentration of 5 mg/mL, and divide it into two equal parts. Add kaempferol to one part, and add an equal volume of DMSO to the other part. After mixing, incubate at room temperature for 1 hour. Then, add various concentrations of the organic solvent mixture and incubate for 30 minutes (for CETSA, heat at a gradient temperature from 37°C to 52°C for 5 minutes). Centrifuge the samples, mix the supernatant with electrophoresis loading buffer, heat to denature, and analyze by Western blotting.

### Immunofluorescence (IF)

Cells are seeded in confocal dishes (NEST, 801002) and cultured overnight. Following drug treatment, they are washed with PBS, fixed with paraformaldehyde, permeabilized with Triton X-100, and blocked with 2.5% BSA. Subsequently, the cells are incubated with the primary antibody and a fluorescently labeled secondary antibody at room temperature. The cells are stained with phalloidin (Proteintech, PF00003) and a fluorescence quencher containing DAPI (Beyotime Biotechnology, P0131-25 mL), and then observed under a laser confocal microscope. For tissue samples, cryosections are prepared using a cryostat microtome. After drying the slides, the experimental procedure follows the same steps as those for the cells.

### Cytoplasmic and nuclear fractionation

After washing with PBS, cells are lysed with cytoplasmic extraction buffer (Invent Biotechnologies, SC-003) to separate the cytoplasmic fraction. This is achieved by centrifugation, after which the supernatant is collected. The pellet is then washed with PBS and lysed with nuclear extraction buffer to extract nuclear proteins. Following washing and centrifugation to separate the nuclear proteins, both the cytoplasmic and nuclear fractions are mixed with electrophoresis loading buffer. They are then heated to denature and analyzed by Western blotting.

### Click-iT metabolic labeling

Cells are cocultured with Click-iT^®^ GalNAz (Thermo Fisher, C33365) for 48 hours. After washing with PBS, the cells are sonicated to lyse them, and the proteins are quantified. For the click reaction, Biotin-PEG4-alkyne (CONFLUORE, BCP-14) detection reagent is mixed with Click-iT reaction buffer (Invitrogen™, C10276), followed by the addition of Copper (II) sulfate and Click-iT reaction buffer additive. The mixture is vortexed and rotated to facilitate the reaction. Finally, glycosylated proteins are enriched using streptavidin-agarose beads, washed, and resuspended. The enriched glycosylated proteins are collected and prepared for subsequent gel electrophoresis analysis.

### Cell transfection

Cells are cultured to reach 70% confluence. According to the Lipofectamine 3000 transfection reagent manual (Thermo Fisher, L3000150), prepare a DNA mixture containing Opti-MEM medium (Gibco, 31-985-070), the target plasmid, and Lipo3000 reagent, as well as a Lipofectamine 3000 mixture containing Opti-MEM medium and Lipofectamine 3000 transfection reagent. Gently add the Lipofectamine 3000 mixture to the DNA mixture, and after thorough mixing, allow it to stand at room temperature for 15 minutes to form the transfection complex. For transfection, remove the old cell culture medium, add the transfection complex following the addition of fresh culture medium, and culture the cells in an incubator for 6-12 hours before replacing the medium with fresh culture medium. Verify the expression level changes of the target protein by Western blotting 48 hours later.

### UDP-Glo™ Glycosyltransferase assay 21-22

To investigate the effects of kaempferol treatment and His-901/Asp-925 mutations of OGT on its enzymatic activity in colorectal cancer HCT116 cells, wild-type OGT and mutant plasmids (His-901/Ala and Asp-925/Ala) were transfected into 4 million HCT116 cells using Lipofectamine 3000 transfection reagent, with a transfection system containing 20 μg plasmid DNA and 50 μL transfection reagent in Opti-MEM medium. After 6 hours of transfection, the medium was replaced with fresh DMEM complete medium, followed by 24 hours of incubation. Cells were then treated with 50 μM kaempferol for 24 hours. Subsequently, cells were washed with PBS and lysed in RIPA lysis buffer supplemented with protease inhibitors. For OGT enzymatic activity detection, wild-type OGT protein was immunoprecipitated using an anti-OGT-specific antibody, while FLAG-tagged His-901 and Asp-925 mutants were immunoprecipitated with an anti-FLAG antibody. Antibody-antigen complexes were incubated with 25 μL Protein A/G magnetic beads (Thermo Fisher, 88803) at 4°C for 3 hours, followed by three washes with RIPA buffer without protease inhibitors. Purified OGT enzymes (wild-type and mutants) were resuspended in 25 μL transferase buffer and enzymatic activity was quantified using the Promega UDP-Glo™ Glycosyltransferase Assay Kit (V6962) to measure UDP release. Enzyme reaction kinetics were analyzed by monitoring dynamic luminescence signals at room temperature using a multifunctional microplate reader, with detection time points at 5, 10, 20, 40, and 60 minutes, and emission wavelengths set between 620 and 665 nm. Enzyme kinetic parameters were calculated via nonlinear regression fitting.

### Statistical analysis

Data were analyzed using GraphPad Prism 9.0 software and are presented as the mean ± SD. For comparisons between two groups, an independent samples t-test was used. For comparisons among three or more groups, a one-way analysis of variance (ANOVA) was employed. *P < 0.05, **P < 0.01, ***P < 0.001, ns, not statistically significant.

## Results

### O-GlcNAcylation was significantly upregulated in CRC tissues and cells and correlated with cancer staging and poor prognosis

**Figure 1:**
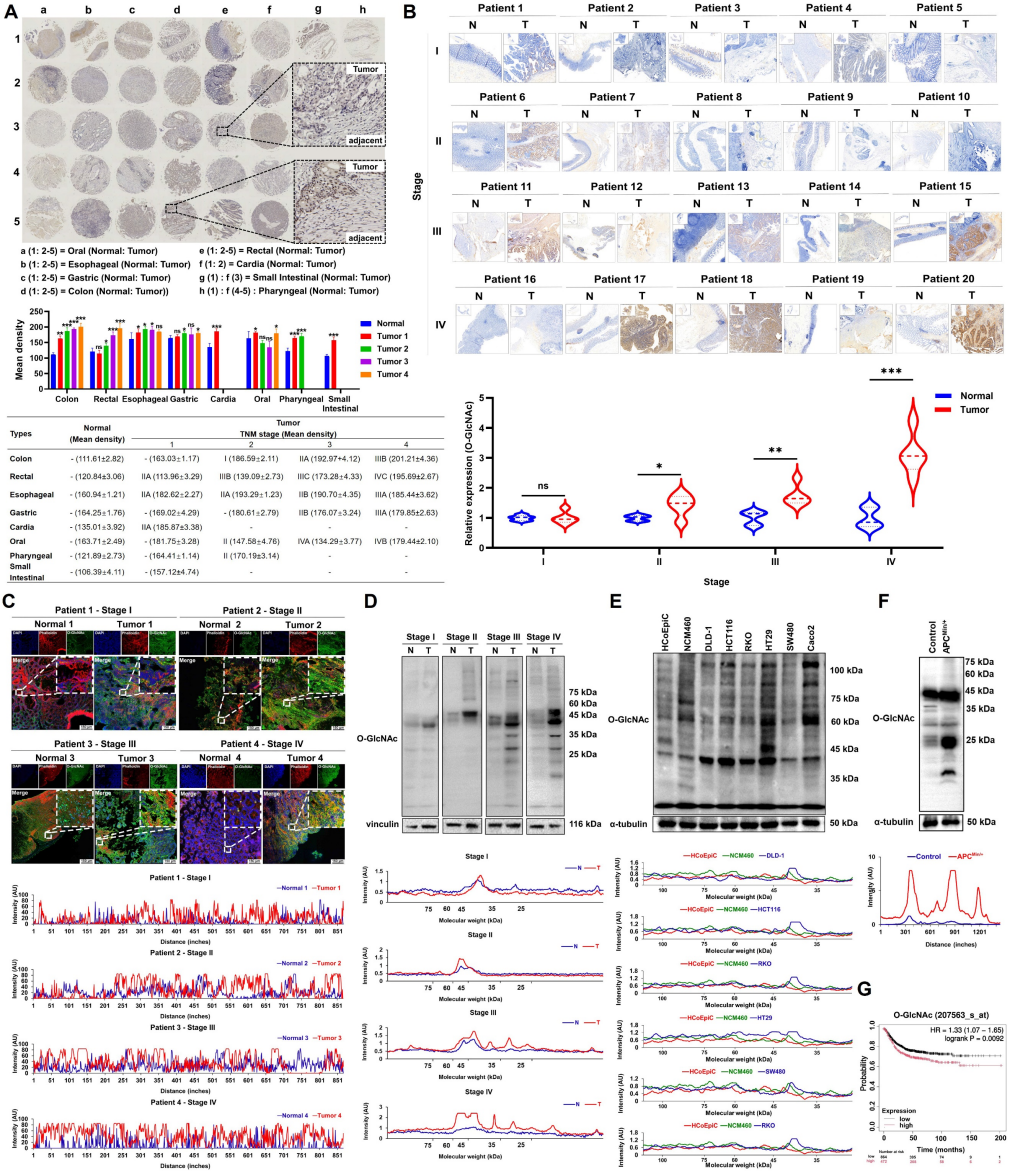
Upregulation of O-GlcNAcylation in CRC tissues and cells. **A,** Analysis of a digestive system tissue microarray. **B**, Immunohistochemical detection of O-GlcNAcylation levels of proteins in cancer and adjacent tissues from 20 clinical CRC tissue samples. **C,** Fluorescence analysis of O-GlcNAcylation of proteins from four CRC patients at different TNM stages. DAPI (blue) marks the nucleus, phalloidin (red) marks the cytoskeleton, and O-GlcNAcylation is shown in green. **D,** Western blotting analysis of O-GlcNAcylation of proteins from four CRC patients at different TNM stages, where N represents adjacent normal tissue and T represents tumor tissue. **E,** O-GlcNAcylation in two colonic epithelial cell lines (HCoEpiC and NCM460) and six CRC cell lines (DLD-1, HCT116, RKO, HT-29, SW480, and Caco2). **F,** Analysis of O-GlcNAcylation in APC^Min/+^ mouse tissues. **G,** Kaplan‒Meier survival curve depicting the overall survival of 1,336 CRC patients stratified by O-GlcNAcylation level, where overall survival was defined as the time from the date of surgery to the date of death or last follow-up. The data are presented as the means ± SDs, * *P* < 0.05, ** *P* < 0.01, *** *P* < 0.001, ns, nonsignificant.

We first analyzed the levels of O-GlcNAcylation in eight types of gastrointestinal tumors using tissue microarray technology. The results showed that the levels of O-GlcNAcylation in colorectal cancer tissues (colon cancer: 201.21 ± 4.36; rectal cancer: 195.69 ± 2.67) were significantly higher than those in other gastrointestinal tumors (oral cancer: 179.44 ± 2.10; esophageal cancer: 185.44 ± 3.62; gastric cancer: 179.85 ± 2.63; cardia cancer: 185.87 ± 3.38; small intestine cancer: 157.12 ± 4.74; pharyngeal cancer: 170.19 ± 3.14; *P* < 0.01). Notably, the O-GlcNAcylation levels in colorectal cancer tissues were significantly higher than those in normal tissues (colon cancer vs. normal tissue: 201.21 ± 4.36 vs. 111.61 ± 2.82; rectal cancer vs. normal tissue: 195.69 ± 2.67 vs. 120.84 ± 3.06; *P* < 0.001). Most importantly, among the eight types of gastrointestinal tumors, only colorectal cancer tissues showed a clear increase in O-GlcNAcylation levels that depended on the TNM stage (from stage I to stage IV, *P* < 0.05;** Figure 1A**). This finding was validated in immunohistochemical analysis of 20 clinical CRC tissue samples (*P* < 0.05; **Figure 1B**). Subcellular localization by immunofluorescence revealed that O-GlcNAc modifications were mainly distributed in the cytoplasm and nucleus, with expression levels progressively increasing with disease progression (**Figure 1C**). Quantitative Western blotting analysis confirmed that O-GlcNAcylation levels were significantly elevated in CRC tissues compared to normal tissues (*P* < 0.01), with modified proteins predominantly enriched in the 10-50 kDa molecular weight range (**Figure 1D**), suggesting that proteins within this range may be key targets of CRC-specific O-GlcNAcylation. *In vitro* cell experiments and the spontaneous CRC model in Apc^Min/+^ mice further demonstrated that tumor cells exhibited an average 83.33% increase in O-GlcNAcylation levels compared to normal colonic epithelial cells (*P* < 0.001), with modified proteins similarly concentrated in the 20-50 kDa range (**Figure 1E-F**). Kaplan-Meier survival analysis indicated that patients with high O-GlcNAcylation levels had significantly lower overall survival rates compared to those with low levels (HR = 1.33,* P* = 0.0092; **Figure 1G**).

### Kaempferol can inhibit the proliferation of CRC both *in vitro* and *in vivo*

**Figure 2:**
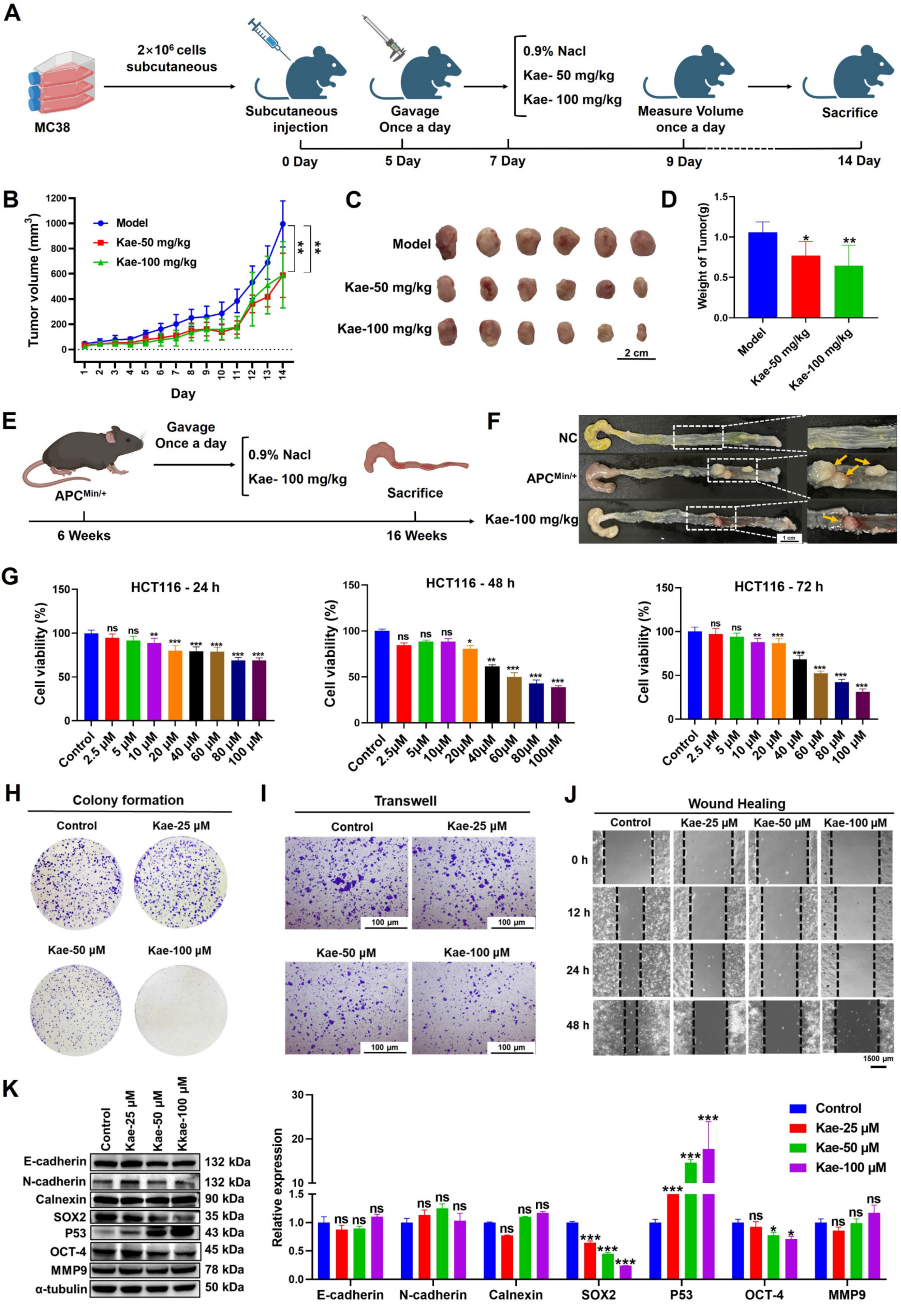
Kaempferol effectively inhibits CRC both *in vitro* and *in vivo*. **A**, MC38 cells were subcutaneously implanted into the right axillary region of C57BL/6 mice to establish a xenograft tumor model, followed by kaempferol treatment; each group in the animal experiment consisted of 6 mice (n=6). **B**, Effect of kaempferol on tumor volume in mice during treatment. **C**, Images of excised tumors from mice. **D**, Tumor weights of the mice. **E**, In the APC^Min/+^ mouse model, kaempferol was administered orally for 10 weeks in the treatment group. **F**, Tumor formation in APC^Min/+^ mice. **G**, The effect of kaempferol on the proliferation of HCT116 cells at 24 h, 48 h, and 72 h was assessed via the sulforhodamine B (SRB) assay.** H**, A colony formation assay was used to evaluate the effect of kaempferol on the stemness of HCT116 cells. **I**, The impact of kaempferol on HCT116 cell migration was examined via a Transwell assay. **J**, Wound healing assay was used to assess the effect of kaempferol on HCT116 cell migration. **K**, Western blotting analysis was performed to detect the influence of kaempferol on proteins related to metastasis, stemness, and proliferation in HCT116 cells. The data are presented as the means ± SDs, * P < 0.05, ** P < 0.01, *** *P* < 0.001, ns indicates no statistically significant difference.

To evaluate whether the anti-CRC efficacy of kaempferol depends on O-GlcNAcylation, an MC38 cell-derived xenograft model and an APC^Min/+^ spontaneous CRC model were established. In the xenograft model, the tumor volume and weight in the treatment group were significantly lower than those in the control group (**Figure 2A-D**). In the APC^Min/+^ mouse model, kaempferol markedly inhibited colorectal adenoma growth (inhibition rate > 70%, **Figure 2E-F**). SRB and colony formation assays revealed that kaempferol significantly inhibited the proliferation of HCT116 cells in a dose- and time-dependent manner (SRB-48 h IC_50_ = 45.3811 μM, **Figure 2G-H**). Additionally, Transwell and wound healing assays revealed that kaempferol significantly reduced the migratory capacity of HCT116 cells (*P* < 0.05, **Figure 2I-J**). Moreover, Western blotting analysis revealed that kaempferol treatment did not affect the expression of epithelial‒mesenchymal transition (EMT) biomarkers, such as E-cadherin, N-cadherin, calnexin, or matrix metalloproteinase-9 (MMP-9). However, kaempferol significantly suppressed the expression of the key transcription factors SOX-2 and OCT-4, which maintain stem cell characteristics, and promoted the expression of the tumor suppressor protein P53 (*P* < 0.05, **Figure 2K**). In summary, kaempferol significantly inhibited CRC progression both *in vitro* and *in vivo*.

### Kaempferol suppresses the expression of O-GlcNAcylation in CRC proteins

**Figure 3:**
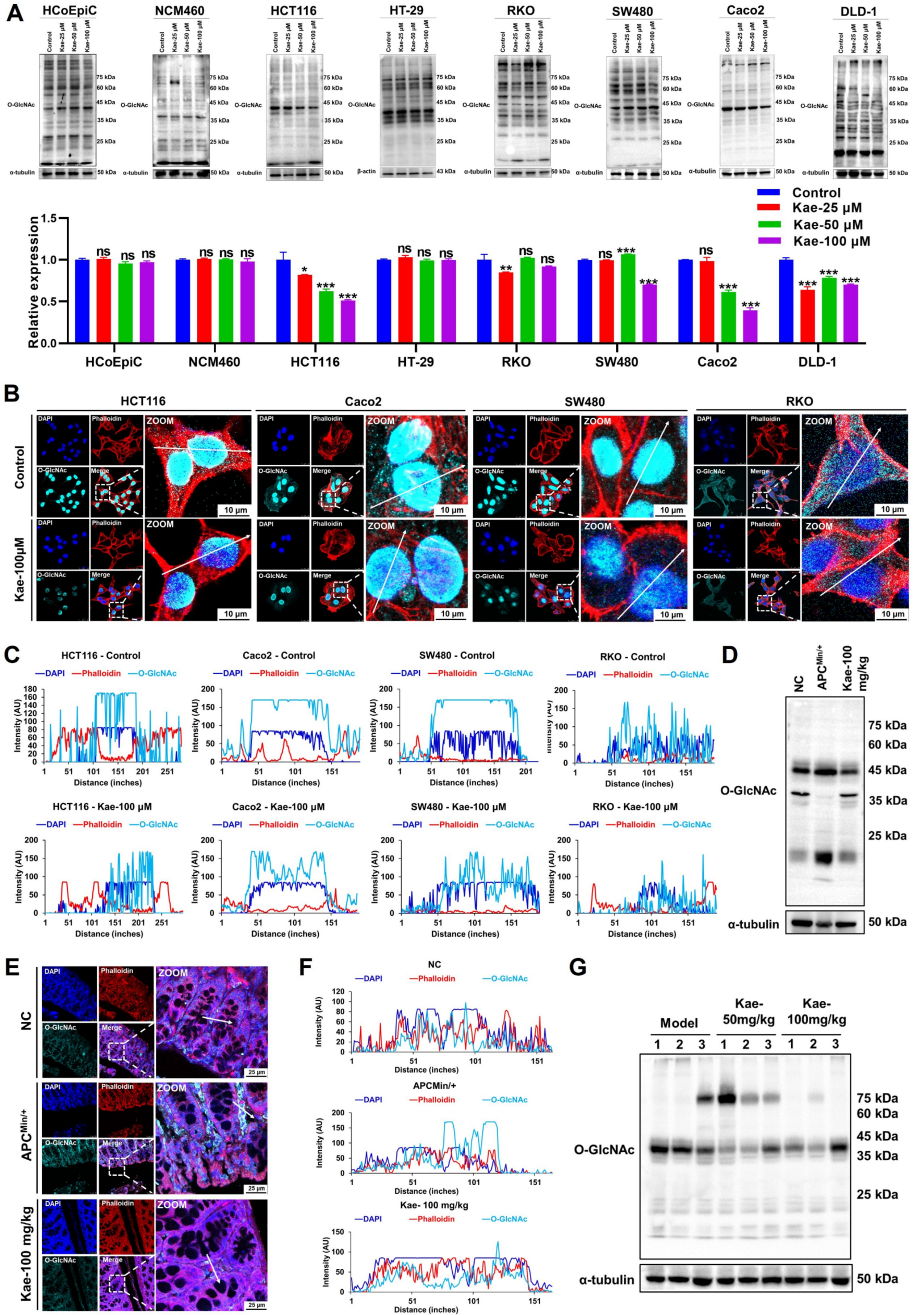
Kaempferol Inhibits protein O-GlcNAcylation in CRC. **A**, Detection and quantification of O-GlcNAcylation levels after treatment with kaempferol (25, 50, 100 µM) in two normal colon epithelial cell lines and six CRC cell lines. **B**, LSCM (laser scanning confocal microscopy) was used to detect the expression and localization of protein O-GlcNAcylation in HCT116, SW480, Caco2, and RKO cells. The nuclear marker DAPI is shown in blue, the cytoskeletal marker phalloidin is shown in red, and O-GlcNAcylation is shown in cyan. **C**, Analysis of the effect of kaempferol on protein O-GlcNAcylation in the four CRC cell lines. **D**, Western blotting analysis of the effect of kaempferol on protein O-GlcNAcylation in the APC^Min/+^ mouse model. **E**, LSCM detection of the effect of kaempferol on the expression and localization of protein O-GlcNAcylation in APC^Min/+^ mice. **F**, Analysis of the effects of kaempferol on protein O-GlcNAcylation in APC^Min/+^ mice via LSCM. **G**, Western blotting analysis of the effects of kaempferol on protein O-GlcNAcylation in the xenograft tumor tissues of mice. The data are presented as the means ± SDs, * P < 0.05, ** P < 0.01, *** *P* < 0.001, ns indicates no statistically significant difference.

To understand the correlation between kaempferol-mediated inhibition of CRC and protein O-GlcNAcylation, we performed Western blotting analysis on two normal colon epithelial cell lines (NCM460 and HCoEpiC) and six CRC cell lines (HCT116, HT29, SW480, RKO, Caco2, and DLD-1) and examined 11 different posttranslational modifications (PTMs). Interestingly, compared with those of the other 10 PTMs, O-GlcNAcylation levels were significantly lower (*P* < 0.05) in five CRC cell lines after kaempferol treatment, with no effect on normal colon epithelial cells (**Figure 3A**; Supplementary Figure 1). Fluorescence analysis further revealed that protein O-GlcNAcylation was predominantly localized in the nucleus of HCT116, SW480, and Caco2 cells (nucleus: cytoplasm = 95.97%: 4.03%), whereas in RKO cells, the distribution was more even (nucleus: cytoplasm = 49.18%: 50.82%). Notably, kaempferol significantly reduced O-GlcNAcylation levels in all four cell lines (**Figure 3B-C**). Additionally, we conducted Western blotting and immunofluorescence analyses on tissue samples from kaempferol-treated APC^Min/+^ model mice. The results indicated that kaempferol significantly decreased O-GlcNAcylation levels in the colorectal tissues of the model group mice (protein molecular weights < 25 kDa and 40-50 kDa; **Figure 3D-F**). Moreover, in the xenograft tumor model, kaempferol markedly reduced O-GlcNAcylation levels in tumor tissues (protein molecular weight 35-45 kDa; **Figure 3G**). Taken together, these findings suggest that kaempferol inhibits CRC growth by reducing intracellular O-GlcNAcylation levels.

### Kaempferol reduces UDP-GlcNAc production and exerts its anti-CRC effects in an OGT-dependent manner

**Figure 4:**
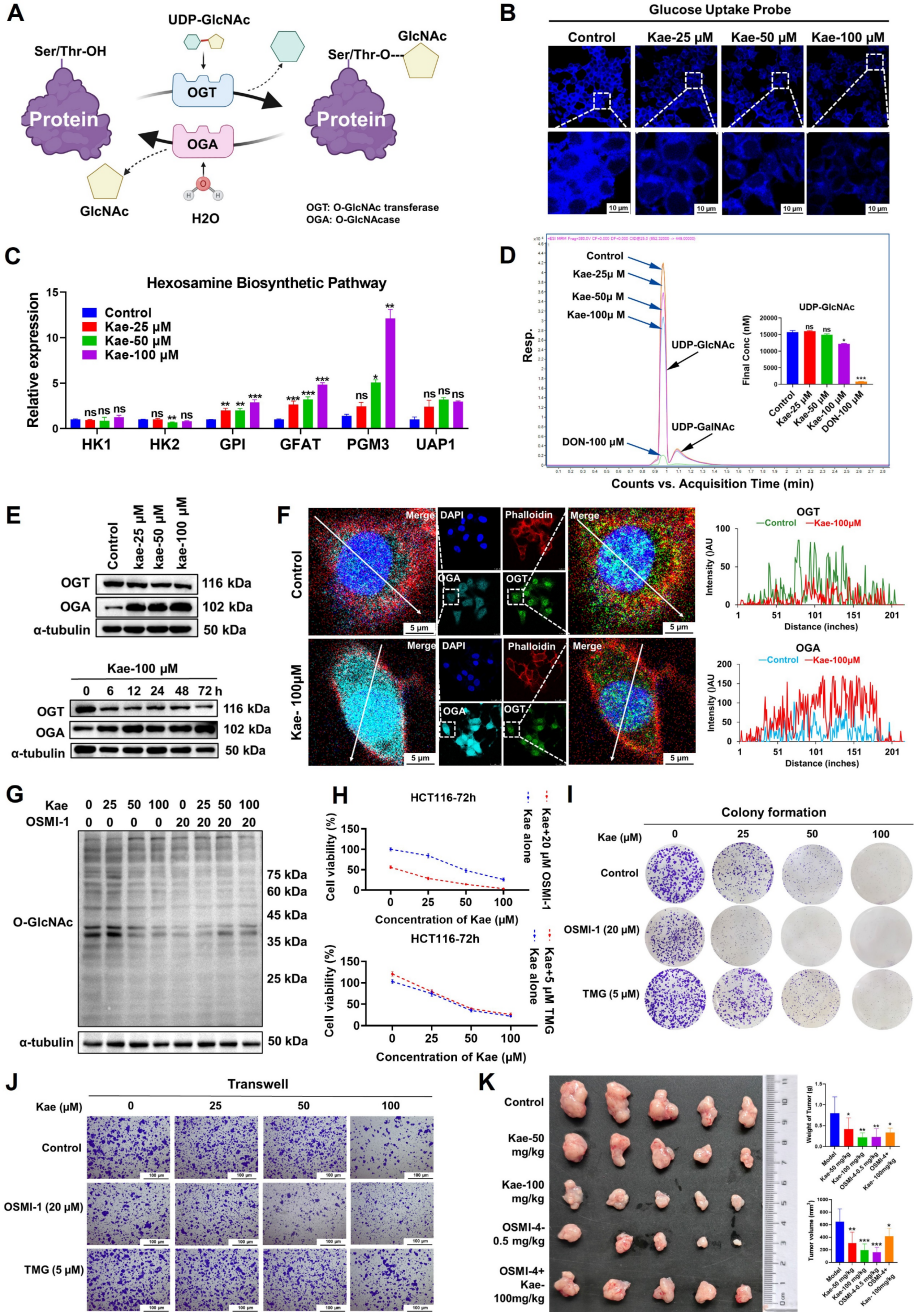
The impact of OGA/OGT on the inhibition of protein O-GlcNAcylation by kaempferol in CRC. **A**, The process of protein O-GlcNAcylation catalyzed by OGT/OGA. **B**, Effects of various concentrations of kaempferol on glucose uptake in HCT116 cells. **C**, Effect of kaempferol on the mRNA expression of HBP-related catalytic enzymes. **D**, LC‒MS analysis of the effect of kaempferol on UDP-GlcNAc levels. **E**, Effects of kaempferol at different concentrations and time points on OGT/OGA expression. **F**, LSCM detection of the effects of kaempferol on OGT/OGA expression and cellular localization. **G**, Effects of various concentrations of kaempferol combined with OSMI-1 on protein O-GlcNAcylation. **H**, Effects of kaempferol combined with OSMI-1 and TMG on CRC cell proliferation.** I**, Colony formation assay to evaluate the effect of kaempferol combined with OSMI-1 and TMG on the colony-forming ability of CRC cells.** J**, Transwell assay to examine the effect of kaempferol combined with OSMI-1 and TMG on CRC cell migration. **K**, The subcutaneous tumor implantation experiment in mice was conducted to examine the effects of kaempferol treatment alone and its combination with the OGT inhibitor OSMI-4 on mouse tumors. The data are presented as the means ± SDs, * P < 0.05, ** P < 0.01, *** *P* < 0.001, ns indicates no statistically significant difference.

UDP-GlcNAc is a key substrate in the hexosamine biosynthetic pathway (HBP), which is primarily catalyzed by β-N-acetylglucosaminyltransferase (OGT) and β-N-acetylglucosaminidase (OGA), leading to the O-GlcNAcylation of proteins (**Figure 4A**). To investigate the molecular mechanism underlying the kaempferol-mediated reduction in protein O-GlcNAcylation, we first examined its impact on glucose uptake in cancer cells, given the critical role of glucose as the primary metabolic substrate for the HBP that governs O-GlcNAcylation dynamics. Subsequent analyses revealed that kaempferol treatment significantly suppressed hexokinase 2 (HK2) protein expression (*P* < 0.05) and accelerated its proteasomal degradation (Supplementary Figure 2A and B), ultimately leading to impaired cellular glucose uptake capacity (**Figure 4B**).

Although the mRNA expression levels of key enzymes in the HBP, including GPI, GFAT, and PGM3, were upregulated with increasing kaempferol concentrations (**Figure 4C**), LC/MS analysis of UDP-GlcNAc levels indicated that kaempferol significantly decreased intracellular UDP-GlcNAc levels (*P* < 0.05, **Figure 4D**). These findings suggest that the kaempferol-induced reduction in protein O-GlcNAcylation may be due to its ability to lower glucose uptake in CRC cells, resulting in decreased UDP-GlcNAc levels. Furthermore, the Western blotting and RT‒PCR results revealed a significant increase in OGT mRNA and protein expression in CRC cells, whereas OGA expression decreased in some cell lines (*P* < 0.05, Supplementary Figure 2C and D). Kaplan-Meier survival analysis showed that patients with high OGT expression had a significantly shorter survival (*P* = 0.016), while no significant correlation was observed between high OGA expression and patient survival (*P* = 0.16, Supplementary Figure 2E). Interestingly, kaempferol downregulated OGT protein expression in a dose- and time-dependent manner but upregulated OGA protein expression (*P* < 0.05, **Figure 4E**, Supplementary Figure 2F). This may be due to kaempferol promoting OGT degradation via the lysosomal pathway while delaying OGA degradation (Supplementary Figure 2G). Additionally, kaempferol significantly increased OGA nuclear localization (control: Kae = 1.00: 2.28) and decreased OGT nuclear localization (control: Kae = 1.00: 0.47; **Figure 4F**).

An investigation of the roles of OGA and OGT in the inhibitory effects of kaempferol on protein O-GlcNAcylation and CRC revealed that inhibiting OGT weakened the ability of kaempferol to reduce protein O-GlcNAcylation (**Figure 4G**), whereas inhibiting OGA enhanced this effect (Supplementary Figure 3A). Furthermore, the results of the SRB assay demonstrated that OGT inhibition diminished the ability of kaempferol to suppress cell proliferation (*P* < 0.05), whereas OGA inhibition did not result in significant differences (**Figure 4H**; Supplementary Figure 2H). However, colony formation, Transwell, and wound healing assays consistently revealed that OGT inhibition weakened the inhibitory effect of kaempferol on CRC cells, whereas OGA inhibition increased this effect (*P* < 0.05, **Figure 4I-J**; Supplementary Figure 2I-L). It is worth noting that in the subcutaneous tumor model in mice, when the OGT-specific inhibitor OSMI-4 was used to inhibit O-GlcNAcylation modification, the antitumor effect of kaempferol not only did not show the expected synergistic effect but instead exhibited a significant antagonistic effect (combination therapy group vs monotherapy group, average tumor volume: 396.5588 mm³ vs 258.2066/172.4777/136.6953 mm³; **Figure 4K**). Collectively, these findings demonstrate that the anti-CRC effects of kaempferol, which are mediated through the suppression of protein O-GlcNAcylation, are mechanistically dependent on OGT functionality.

### Kaempferol targets OGT to inhibit protein O-GlcNAcylation and CRC

**Figure 5:**
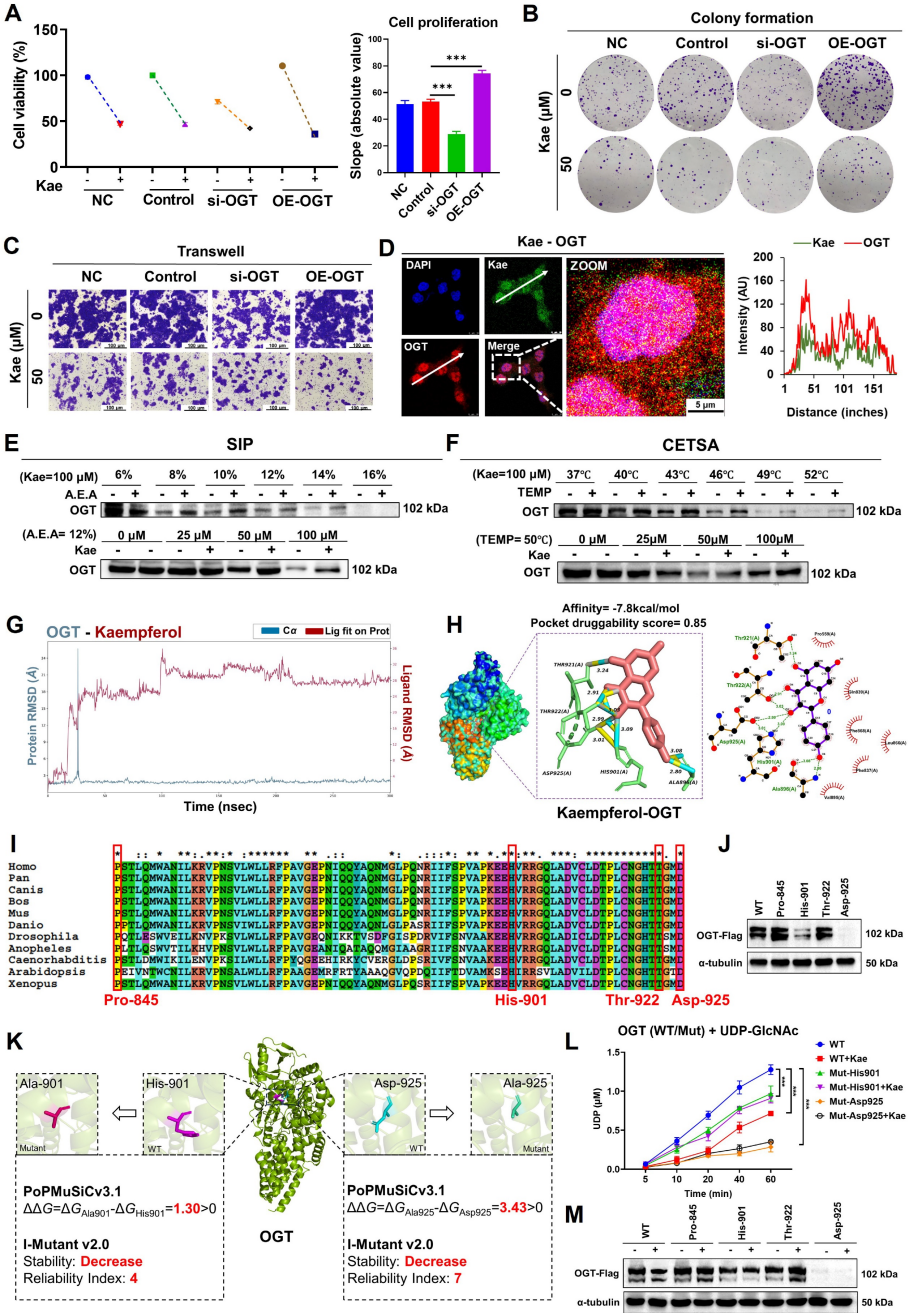
Kaempferol Targets OGT to Exert its Anti-CRC Effects. **A**, Effects of OGT knockdown and overexpression on kaempferol-mediated inhibition of HCT116 cell proliferation. **B**, Effects of OGT knockdown and overexpression on kaempferol-mediated inhibition of HCT116 cell stemness. **C**, Effects of OGT knockdown and overexpression on kaempferol-mediated inhibition of HCT116 cell migration. **D**, Fluorescence colocalization of kaempferol and OGT; DAPI labels the nucleus in blue, kaempferol shows intrinsic green fluorescence, and OGT is marked in red. **E**, SIP assay to detect the binding between kaempferol and OGT. **F**, CETSA confirming the interaction between kaempferol and OGT. **G**, Root mean square deviation (RMSD) of kaempferol and OGT during the 300 ns simulation. The RMSD measures the average displacement of selected atoms in a given frame relative to a reference frame, providing insights into protein structural dynamics and equilibrium during the simulation. For globular proteins, deviations within 1-3 *Å* are acceptable. **H**, Molecular docking between kaempferol and OGT. **I**, Conservation analysis of OGT amino acids. **J**, OGT protein expression after amino acid site mutation. **K,** The effects of the mutations on OGT stability were computed via PoPMusic 2.1 and I-mutant 3.0. **L,** Effects of mutations at the detection site on OGT enzyme activity as analyzed via the UDP-Glo™ glycosyltransferase assay kit. **M**, Effects of OGT amino acid site mutation on the ability of kaempferol to inhibit OGT expression. The data are presented as the means ± SDs, * P < 0.05, ** P < 0.01, *** *P* < 0.001, ns indicates no statistically significant difference.

To investigate the specific role of OGT in the inhibitory effect of kaempferol on CRC, we conducted OGT knockdown and overexpression experiments. SRB assays revealed that the inhibitory effect of kaempferol on HCT116 cell proliferation was attenuated when OGT was knocked down and enhanced when OGT was overexpressed (*P* < 0.05) (**Figure 5A**). The results from the colony formation and Transwell assays further supported the role of OGT (*P* < 0.05) (**Figure 5B-C**, Supplementary Figure 2M and N). However, when OGA was knocked down, the inhibitory effect of kaempferol on the proliferation and migration of HCT116 cells was enhanced (Supplementary Figure 3B-D). Moreover, the fluorescence colocalization results suggested a potential interaction between kaempferol and OGT (**Figure 5D**; kaempferol and OGA in Supplementary Figure 3E). Subsequent SIP and CETSA experiments confirmed the binding relationship between kaempferol and OGT (Kae = 100 μM, A.E.A. = 12%-14%, TEMP = 46 - 52°C) (**Figure 5E**-**F**; kaempferol and OGA in Supplementary Figure 3F and G).

To elucidate the binding mode between kaempferol and OGT, a 300 ns molecular dynamics simulation using Desmond was performed. The simulation revealed that kaempferol's root mean square deviation (RMSD) value initially increased rapidly and reached equilibrium at 30 ns. The RMSD value of OGT spiked at 20 ns before returning to its initial state indicated a potential positional shift during the simulation (**Figure 5G**; kaempferol and OGA in Supplementary Figure 3H). Additionally, the OGT root mean square fluctuation (RMSF) values ranged from 0.6-4.8, with the greatest fluctuations observed in the 500--600 residue region, suggesting significant conformational changes in this area during the simulation (Supplementary Figure 4A; kaempferol and OGA in Supplementary Figure 3I). Notably, the interaction between kaempferol and OGT during the simulation highlighted key amino acid residues, including Trp-469, Asp-473, Pro-845, Gln-849, Asn-853, Lys-856, Tyr-879, Asn-882, and Met-883 (Supplementary Figure 4B; kaempferol and OGA in Supplementary Figure 3J).

Molecular docking via AutoDock Vina 1.2.2 revealed strong binding affinity between kaempferol and OGT (binding energy = -7.8 kcal/mol), whereas DoGSiteScorer predicted high druggability for OGT (score = 0.85). The key binding sites included Asp-925, His-901, Ala-896, Thr-922, and Thr-921 (**Figure 5H**; kaempferol and OGA in Supplementary Figure 3K). Amino acid conservation analysis revealed that Pro-845, His-901, Thr-922, and Asp-925 were highly conserved, indicating their critical role in maintaining OGT structure and function (**Figure 5I**). Mutations at these four sites were introduced, and the His-901 and Asp-925 mutations significantly downregulated OGT expression (**Figure 5J**, Supplementary Figure 4C). Further analysis using PoPMuSiC 2.1 and I-Mutant 3.0 was conducted to evaluate the effects of mutations at His-901 and Asp-925 on the stability of OGT [[xxiii]-[xxiv]]. The results demonstrated that His-901 and Asp-925 are located near the catalytic active center of OGT. Mutations at these residues may directly impair OGT's catalytic activity and substrate binding affinity, leading to reduced intracellular stability and accelerated degradation (**Figure 5K**).

To determine the effects of His-901 and Asp-925 mutations in the OGT protein and kaempferol treatment on OGT enzymatic activity in HCT116 colorectal cancer cells, we employed the UDP-Glo™ glycosyltransferase assay kit. Quantitative analysis revealed that 50 μM kaempferol treatment for 48 hours significantly inhibited the glycosyltransferase activity of wild-type OGT (*P* < 0.001), suggesting direct binding and enzymatic suppression by kaempferol. Strikingly, site-directed mutagenesis of His-901 and Asp-925 to alanine substantially compromised OGT catalytic function: the His-901 mutant retained 70-80% of the wild-type UDP release level, whereas the Asp-925 mutant exhibited markedly reduced activity (15-25% of that of the wild type) (**Figure 5L**) (*P* < 0.001). Further Western blotting and SRB assays revealed that His-901 and Asp-925 mutations diminished the ability of kaempferol to suppress OGT expression and inhibit cellular proliferation (**Figure 5M**, Supplementary Figure 4D). These findings collectively establish His-901 and Asp-925 as essential structural determinants mediating both OGT enzymatic activity and pharmacological targeting by kaempferol.

### Identification of the downstream O-GlcNAcylation target Hsp47

**Figure 6:**
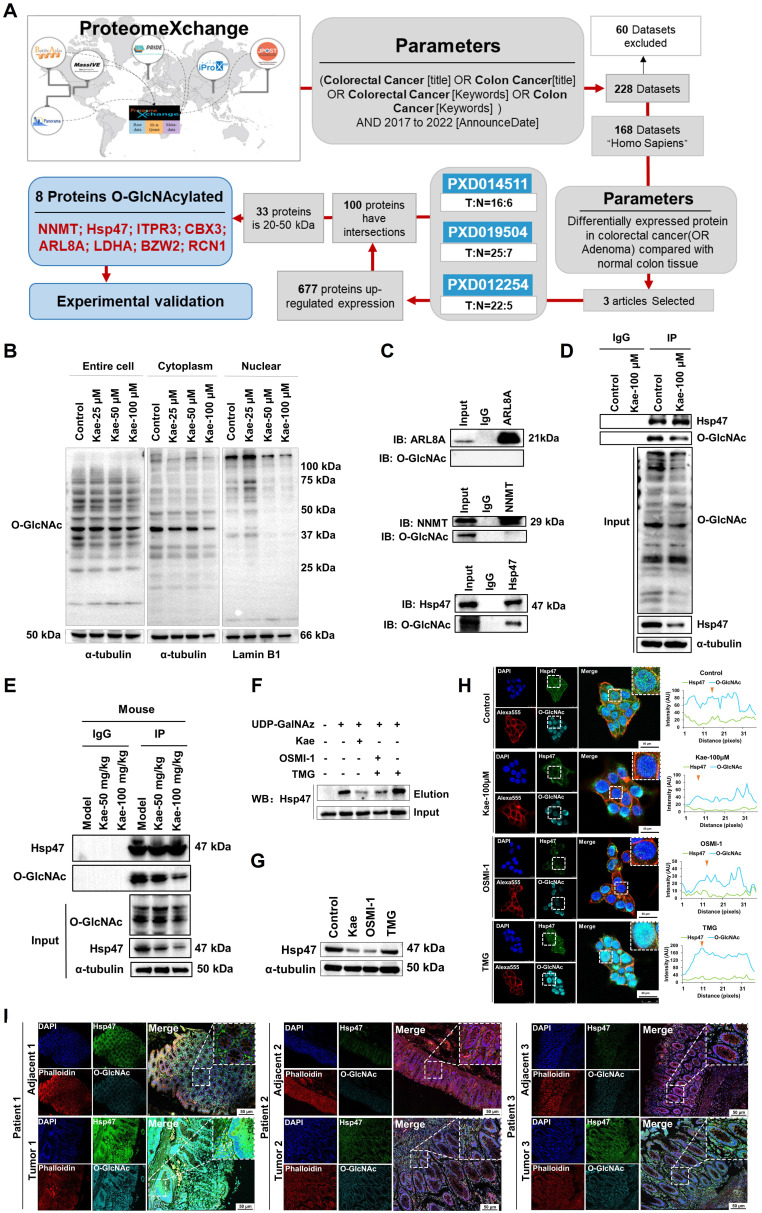
Kaempferol Reduces O-GlcNAcylation of Hsp47. **A**, Bioinformatics analysis and screening workflow for O-GlcNAcylated proteins in CRC. **B**, Effect of kaempferol on protein O-GlcNAcylation in the cytoplasm and nucleus of HCT116 cells. **C**, Immunoprecipitation validation of glycosylations in NNMT, Hsp47, and ARL8A. **D**, Immunoprecipitation analysis of the effect of kaempferol on Hsp47 glycosylation. **E**, Verification of the effect of kaempferol on the glycosylation of Hsp47 at the animal level. **F**, Click-iT enzyme labeling enrichment assay to evaluate the effect of kaempferol on Hsp47 glycosylation. **G**, Effect of kaempferol on Hsp47 protein expression. **H**, LSCM analysis of the effect of kaempferol on Hsp47 expression. The nuclear marker DAPI is shown in blue, Hsp47 in green, the cytoskeletal marker phalloidin in red, and O-GlcNAcylation in cyan.** I**, LSCM analyzes the expression of Hsp47 in cancer and matched adjacent non-cancerous tissues.

Research shows that O-GlcNAcylation of proteins can reduce their degradation by inhibiting the ubiquitination pathway, thereby stabilizing protein expression levels [[xxv]-[xxvi]]. To explore the O-GlcNAcylated proteins inhibited by kaempferol, we utilized the ProteomeXchange database (https://www.proteomexchange.org/) and identified three human datasets related to "colorectal cancer" or "colon cancer" from 2017-2022: "PXD012254," "PXD019504," and "PXD014511." Further bioinformatics analysis revealed 677 proteins that were highly expressed in CRC, 33 of which had molecular weights between 20 and 50 kDa (Table 2). Five O-GlcNAcylation site databases were used: the O-GlcNAc Database v2.0 (https://www.oglcnac.Mcw.edu/), YinOYang-1.2 (https://services.healthtech.dtu.dk/services/YinOYang-1.2/), GPP (https://comp.chem.nottingham.ac.uk/cgi-bin/glyco/bin/getparams.cgi), O-GlcNAcAtlas (https://www.oglcnac.org/atlas/search/) and O-GlcNAcPRED-DL (https://oglcnac.org/pred_dl/input_fasta), combined with survival analysis and subcellular localization data from UniProt (Table 3), eight candidate proteins were selected for experimental validation (**Figure 6A**; Table 4).

Through nuclear‒cytoplasmic fractionation and Western blotting analysis, we found that the O-GlcNAcylation levels of 20-50 kDa proteins changed significantly in the cytoplasm but not in the nucleus (**Figure 6B**). On the basis of these findings, we selected NNMT, SERPINH1, and ARL8A for immunoprecipitation validation and discovered glycosylation on Hsp47, which is encoded by SERPINH1 (**Figure 6C**). Further immunoprecipitation and Click-iT enzyme labeling enrichment experiments confirmed that kaempferol reduced both the glycosylation level and protein expression of Hsp47 (**Figure 6D-F**). To fully understand the expression of Hsp47 in CRC and its relationship with O-GlcNAcylation, Western blotting analysis revealed that Hsp47 was highly expressed in cancer cells (Supplementary Figure 5A), and kaempferol treatment significantly reduced its expression (**Figure 6G-H**). LSCM analysis revealed that Hsp47 was primarily localized in the cytoplasm, with its expression being significantly higher in cancer tissues than in matched adjacent noncancerous tissues. Additionally, elevated expression of Hsp47 was associated with increased protein O-GlcNAcylation (**Figure 6I**). Clinically, Hsp47 overexpression correlated with reduced overall survival rates (HR = 1.6, *P* = 0.044) in the cancer cohort, further validating its prognostic significance (Supplementary Figure 5B).

### The biological function of Hsp47

**Figure 7:**
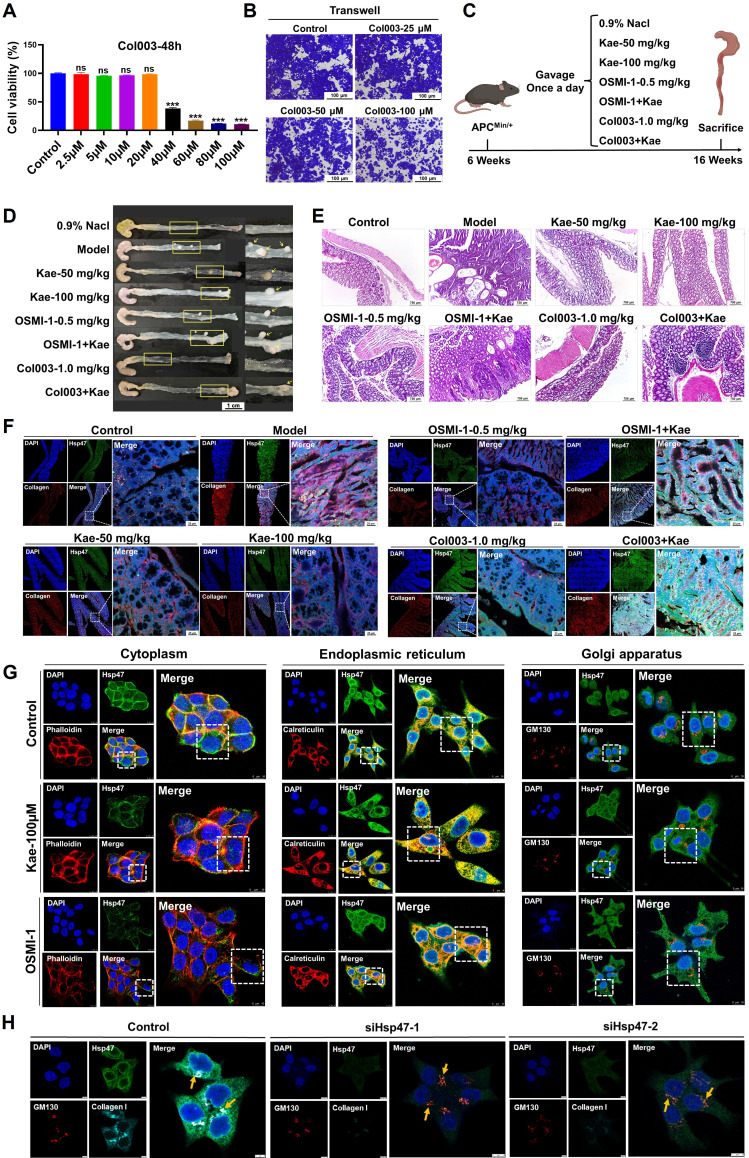
The role of Hsp47 in kaempferol-mediated inhibition of CRC. **A**, SRB assay assessing the effect of Col003 on the proliferative activity of CRC cells.** B**, Transwell assay evaluating the impact of Col003 on the migration of CRC cells. **C**, In the APC^Min/+^ mouse model, the treatment groups included oral administration of 50 mg/kg and 100 mg/kg of kaempferol, intraperitoneal injection of 0.5 mg/kg OSMI-1 and 1.0 mg/kg CoI003, as well as the combined use of kaempferol with OSMI-1 and CoI003, administered for 10 weeks.** D**, Tumor formation in APC^Min/+^ mice. **E**, HE staining was used to evaluate the pathological features of the colonic tissues of the animals. **F**, LSCM detection of Hsp47 and collagen I expression in mouse tissues; DAPI was used to label the nuclei in blue, collagen I in red, and Hsp47 in green. **G**, LSCM detection of the effect of kaempferol on the localization of Hsp47 within the cytoplasm, endoplasmic reticulum, and Golgi apparatus; DAPI is used to label the nuclei in blue, and the cytoplasm, endoplasmic reticulum, and Golgi apparatus are marked in red (phalloidin, calnexin, GM130), with Hsp47 in green. **H**, LSCM detection of the effect of Hsp47 knockdown on the localization of collagen I in the Golgi apparatus. The data are presented as the means ± SDs, * P < 0.05, ** P < 0.01, *** *P* < 0.001, ns indicates no statistically significant difference.

Col003 is a selective and potent inhibitor of Hsp47 that competitively binds to the collagen-binding site on Hsp47. Disruption of the stability of the collagen triple helix effectively inhibited collagen secretion. The results from the Transwell and SRB assays indicated that Col003 significantly reduced the migratory capacity and proliferative activity of cancer cells (*P* < 0.05; **Figure 7A-B**). In the APC^Min/+^ mouse model, the combined administration of Col003 and kaempferol reduced the efficacy of kaempferol alone (**Figure 7C-D**). Furthermore, HE staining revealed that the combination of kaempferol with OGT and Hsp47 inhibitors did not markedly ameliorate atrophy or structural distortion of the colonic mucosal epithelium, crypts, or muscularis mucosa in mice compared with the effects of monotherapy with kaempferol or OGT/Hsp47 inhibitors alone. However, the combination still exhibited certain therapeutic efficacy relative to the model group (**Figure 7E**). LSCM detection showed that both kaempferol and OSMI-1 inhibited Hsp47 expression and reduced the secretion of type I collagen in tissues; however, compared to the monotherapy groups, when kaempferol was combined with OSMI-1 or Col003, this effect was not further enhanced. On the contrary, the expression of type I collagen in colon tissues increased, and the anti-CRC efficacy decreased (**Figure 7F**).

Further analysis showed that treatment with kaempferol and OSMI-1 reduced the overall intracellular distribution of Hsp47, particularly its accumulation in the cytoplasm (kaempferol: reduced by 64.28%; OSMI-1: reduced by 91.39%), while increasing its retention in the endoplasmic reticulum (kaempferol: increased by 12.04%; OSMI-1: increased by 15.21%), with minimal impact on Golgi accumulation (**Figure 7G**). Furthermore, to verify the ability of Hsp47 to independently regulate phenotypes, we constructed an Hsp47 knockdown cell model. The results showed that Hsp47 knockdown significantly inhibited CRC migration (the scratch assay showed a decrease in cell number, Edu assay showed reduced cell proliferation, and the colony formation assay showed reduced cell stemness) (Supplementary Figure 5C-E). It is worth noting that immunofluorescence results show that Hsp47 knockdown can induce a reduction in the localization of intracellular collagen I in the Golgi apparatus, delaying its secretion (**Figure 7H**). The above findings indicate that Hsp47, as a central regulator of collagen secretion, can be retained in the endoplasmic reticulum by kaempferol, hindering the transport of pro-collagen I to the Golgi apparatus, thereby delaying the maturation and secretion of collagen I, and ultimately inhibiting CRC.

### The O-GlcNAc modification site Ser76 on Hsp47 is crucial for the CRC inhibitory effect of kaempferol

**Figure 8:**
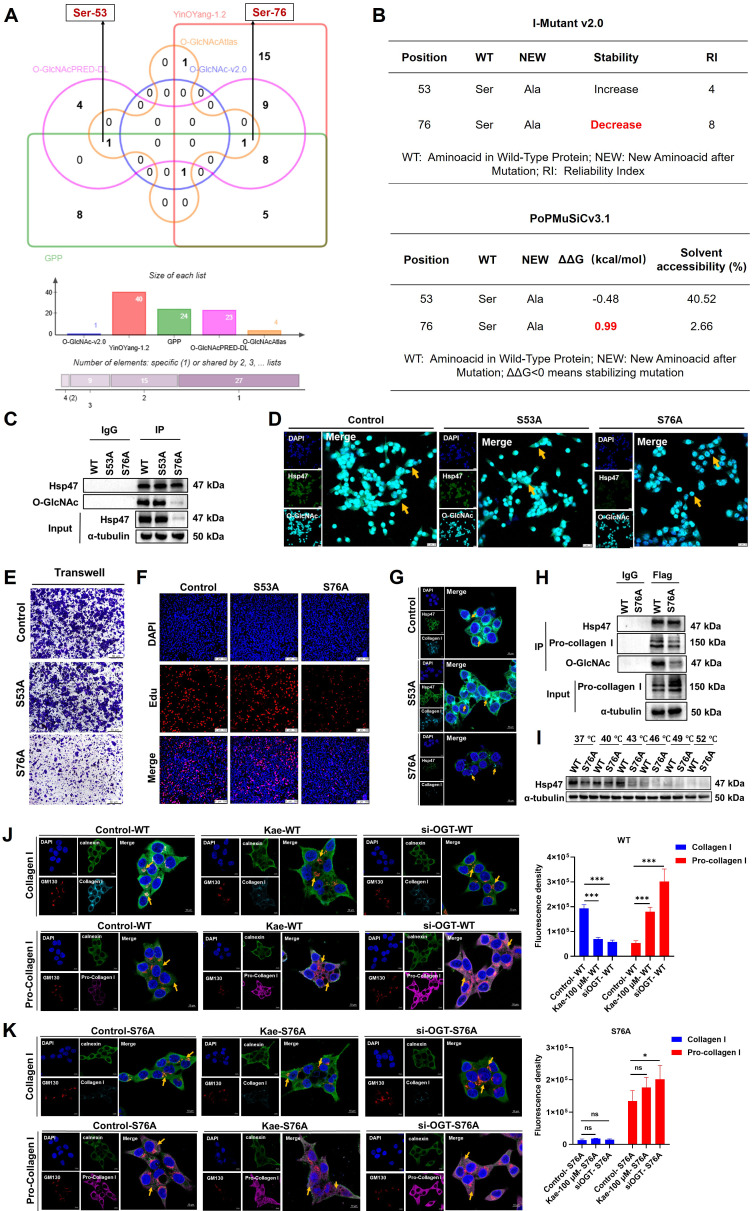
The O-GlcNAc modification site Ser76 on Hsp47 is crucial for the CRC inhibitory effect of kaempferol. **A**, Prediction of O-GlcNAcylation sites on Hsp47 via the O-GlcNAc Database v2.0, YinOYang-1.2, GPP, O-GlcNAcAtlas, and O-GlcNAcPRED-DL. **B**, Prediction of protein stability after mutation at potential glycosylation sites via PoPMuSiC 2.1 and I-Mutant 3.0. **C**, Immunoprecipitation to verify the effects of Hsp47 mutants S53A and S76A on the O-GlcNAcylation of Hsp47 protein. **D**, Immunofluorescence detection of the effects of Hsp47 mutants S53A and S76A on the O-GlcNAcylation of the Hsp47 protein. **E**, The Transwell experiment verifies the effect of Hsp47 mutants S53A and S76A on cell migration. **F**, Edu experiment verifies the effect of Hsp47 mutants S53A and S76A on cell proliferation. **G**, Immunofluorescence validation of the effects of Hsp47 mutants S53A and S76A on collagen I secretion function in cells. **H**, Western blotting verifies the functional impact of the mutant Ser76 on the interaction between Hsp47 and pro-collagen. **I**, CETSA verifies the effect of the S76A mutant on the thermal stability of Hsp47. **J**, Immunofluorescence analysis was performed to investigate the distribution of collagen I and procollagen I within the endoplasmic reticulum and Golgi apparatus in cells treated with kaempferol or OGT knockdown. **K**, Immunofluorescence analysis demonstrated that the blockade of O-GlcNAcylation at the Ser-76 site on Hsp47 inhibits kaempferol-mediated secretion of collagen type I. The data are presented as the means ± SDs, * P < 0.05, ** P < 0.01, *** *P* < 0.001, ns indicates no statistically significant difference.

To elucidate the molecular mechanism of Hsp47 O-GlcNAcylation modification in the inhibition of CRC by kaempferol, this study systematically identified the key O-GlcNAc modification sites of Hsp47 and their functional significance. Analysis of the predicted site revealed two potential O-GlcNAc modification sites on Hsp47 (Ser53 and Ser76) (**Figure 8A**). Further predictions of protein stability were conducted using PoPMuSiC 2.1 and I-Mutant 3.0 software. The results showed that the Ser76 site mutation (ΔΔG = 0.99 kcal/mol, RI = 8) significantly reduced the structural stability of Hsp47, while the Ser53 site mutation had a smaller impact (**Figure 8B**). To verify this prediction, we constructed Hsp47 S53A and S76A mutant plasmids and overexpressed them in HCT116 cells. Immunoprecipitation and Western blotting analysis revealed that the O-GlcNAc modification level of the S76A mutant was much lower, while the S53A mutant showed no noticeable change (**Figure 8C**). This result was further confirmed by immunofluorescence experiments. (**Figure 8D**). Functional studies indicate that the S76A mutation significantly inhibits the migration (Transwell assay) and proliferation (EdU assay) of HCT116 cells (**Figure 8E-F**). LSCM detection showed that collagen I secretion was inhibited in HCT116 cells with the S76A mutant, while no such phenomenon was observed in the wild type (WT) and S53A groups (**Figure 8G**). Co-IP experiments confirmed that the binding efficiency of the S76A mutant to procollagen I was reduced (**Figure 8H**). CETSA analysis shows that the thermal stability of the S76A mutant significantly decreases (**Figure 8I**), indicating that O-GlcNAc modification at the Ser76 site is key to preserving the structural stability and function of Hsp47. To confirm that Ser76 O-GlcNAc modification is a key target for kaempferol in regulating collagen metabolism, we demonstrated that blocking this modification can eliminate the biological effects of kaempferol. Normally, in healthy cells, procollagen moves from the endoplasmic reticulum (ER, marked by Calnexin) to the Golgi apparatus (GA, marked by GM130) for processing, with the help of Hsp47. Our experiments found that treatment with kaempferol (100 μM) or OGT knockdown can cause pro-collagen to be retained in the endoplasmic reticulum (increased co-localization with calnexin) and reduce the accumulation of collagen I in the Golgi apparatus (decreased co-localization with GM130). However, in S76A mutant cells, compared to the control group, even with high-dose kaempferol treatment (100 μM) or combined OGT knockdown, there were no significant changes in collagen I secretion levels or procollagen transport (**Figure 8J-K**). These results indicate that kaempferol regulates the affinity of Hsp47 for procollagen by reducing O-GlcNAc modification at the Ser76 site, thereby affecting the secretion process of collagen I.

## Discussion

In this study, we elucidated that kaempferol targets specific amino acid residues on OGT, namely, His-901 and Asp-925, significantly reducing its protein expression and stability. Kaempferol concurrently downregulates HK2 protein expression, inhibiting the uptake of glucose by colorectal cancer (CRC) cells and consequently decreasing the levels of the HBP pathway substrate UDP-GlcNAc. These mechanisms lead to a decrease in the O-GlcNAcylation levels of the downstream target protein Hsp47, which further reduces its expression and alters its localization within the endoplasmic reticulum, impairing the transport and maturation of collagen I to the Golgi apparatus. These effects are ultimately key factors involved in the ability of kaempferol to inhibit CRC growth and metastasis. Therefore, our research establishes for the first time the relationship between collagen-related proteins in the extracellular matrix (ECM) and O-GlcNAcylation, clarifying that kaempferol inhibits CRC growth and metastasis through this mechanism, thus providing new insights for the treatment of colorectal cancer in humans.

O-GlcNAcylation is increased in CRC tissues and cells and is associated with cancer stage and poor prognosis. Early studies have indicated that protein O-GlcNAcylation primarily occurs in normal human organs such as the brain and liver 27; however, its specific distribution in the gastrointestinal tract and its role in carcinogenesis remain unclear. Through tissue microarray analysis, we observed that the level of protein O-GlcNAcylation in CRC is significantly greater than that in other types of gastrointestinal cancers. Additionally, patients with different TNM stages exhibited a marked increase in protein O-GlcNAcylation, particularly in CRC. However, the current microarray sample size is small, which is one of the limitations of the study. In the future, we will collect different numbers of cancer cases to expand the cohort. Meanwhile, in order to enhance the reliability of the conclusion that glycosylation level is only related to the TNM stage of colorectal cancer, we additionally collected tissue specimens from 20 patients with colorectal cancer, including 5 cases in each TNM stage (I, II, III, IV). After immunohistochemical detection of O-GlcNAcylation in this batch of samples, the results further verified the preliminary findings. Kaplan-Meier survival analysis indicated that patients with high levels of O-GlcNAcylation had poorer prognoses, suggesting that reducing protein O-GlcNAcylation in CRC may be an effective strategy for inhibiting tumor growth. While early studies have shown that specific tannin polyphenols can effectively inhibit the activity of ppGalNAc-Ts and decrease intracellular O-GlcNAc levels, thereby suppressing the migration and invasion of CRC cells, the relationship between kaempferol, a polyphenolic compound, and protein O-GlcNAcylation remains unclear. In our study, we found that kaempferol significantly reduces protein O-GlcNAcylation levels in mouse tumor tissues and CRC cells while effectively inhibiting CRC growth and metastasis. Interestingly, we also observed variations in the distribution of O-GlcNAcylation among different CRC cell lines. In HCT116, Caco2, and SW480 cells, O-GlcNAcylation was primarily concentrated in the nucleus, with limited distribution in the cytoplasm. However, in RKO cells, there was no significant difference in O-GlcNAcylation levels between the nucleus and cytoplasm, which may be related to specific regulatory factors that differ among cell lines.

Kaempferol, a natural flavonoid compound, has attracted significant attention in cancer research because of its multitarget regulatory properties. It exerts broad-spectrum antitumor effects by inhibiting cell proliferation, inducing apoptosis, regulating oxidative stress, and blocking key carcinogenic pathways, such as the PI3K/AKT/mTOR, NF-κB, and STAT3 pathways. In recent years, research on this topic in colorectal cancer has been particularly in depth. For example, kaempferol can inhibit colorectal cancer stem cell self-renewal by downregulating the Wnt/β-catenin signaling pathway and reversing tumor drug resistance through epigenetic regulation (e.g., miRNA- 21 inhibition). This property has been validated *in vitro* (50-100 μM) and in animal models (50-100 mg/kg) without showing significant organ toxicity. For example, Pu et al. (2024) confirmed that 100 μM kaempferol selectively induces apoptosis in colorectal cancer cells without affecting normal intestinal epithelial cells 28. Furthermore, Yu et al. (2024) demonstrated that 50-100 mg/kg kaempferol significantly inhibits the metastasis of gastrointestinal cancer, confirming its dosage rationale and safety 29. However, the potential of combining kaempferol with existing CRC therapies has yet to be explored. Based on previous work confirming that kaempferol has unique intestinal pharmacokinetic characteristics—maintaining high concentrations and prolonged retention in colonic tissue through enterohepatic circulation 30-31 and the high recurrence rates of 15-50% after existing CRC therapies, the lack of improvement in survival for early-stage patients with oxaliplatin, and the inability of targeted drugs like Edrecolomab/Bevacizumab to significantly enhance overall survival 32. Kaempferol, as a natural low-toxicity compound, shows a significant negative correlation with CRC incidence. Through multidimensional analysis, we have confirmed its synergistic effect with the chemotherapeutic drug oxaliplatin: SRB/Edu proliferation assays showed that the combination of 50 μM kaempferol and 5 μM oxaliplatin had a combination index (CI) of 0.8025 (Chou-Talalay method), indicating a significant synergistic anti-proliferative effect (CI<1) (Supplementary Figure 6A-B). The Transwell migration and colony formation assays further revealed that the combination therapy significantly enhanced the migration and stemness inhibition rates compared to the monotherapy group, demonstrating its effectiveness in blocking the metastatic process (Supplementary Figures 6C-D), providing a new paradigm for CRC combination therapy.

In the mechanistic study, we found that kaempferol reduced the level of UDP-GlcNAc, the substrate of the hexosamine biosynthesis pathway (HBP), and exerted its anti-CRC effect in an OGT-dependent manner. Specifically, kaempferol reshapes the HBP metabolic network in a bidirectional manner by promoting HK2 degradation (proteasome pathway) and upregulating GPI/GFAT/PGM3 (transcriptional feedback). On the one hand, it reduced the upstream driving force of UDP-GlcNAc synthesis (glucose uptake by HK2 and CRC cells), and on the other hand, it enhanced the downstream metabolic flow (GFAT/PGM3), which ultimately led to a reduction in the cellular UDP-GlcNAc pool, further limiting the availability of OGT substrates. In contrast to the findings of Wu et al 33, who reported that melatonin downregulates GFAT to reduce intracellular UDP-GlcNAc levels, the inhibition of the upstream enzyme HK2 by kaempferol has a broader impact on UDP-GlcNAc flux within the HBP. In addition, we explored the effects of kaempferol on glycosyltransferases OGT and OGA. Notably, kaempferol significantly decreased the expression and stability of OGT protein, while increased the expression of OGA protein, and these regulatory effects were concentration- and time-dependent. Subsequent molecular docking simulation, SIP and CETSA experiments confirmed the strong binding interaction between kaempferol and the two enzymes. Importantly, in cellular phenotypic experiments, we observed that inhibition or knockdown of OGT reduced the anti-CRC effect of kaempferol, while its overexpression enhanced the inhibitory effect of kaempferol on CRC. In contrast, OGA inhibition and knockdown enhanced the anti-CRC effect of kaempferol, which we believe stems from the compensatory vulnerability state after OGA inhibition - TMG-induced O-GlcNAc over-accumulation leads to endoplasmic reticulum stress 34 and energy crisis 35, making cells more sensitive to OGT-targeted inhibition of kaempferol. Further binding experiments indicate that kaempferol interacts with specific sites on OGT, including His-901 and Asp-925. These residues do not overlap with the classic UDP-GlcNAc binding sites (His498, His558, Asp554, Lys842, Thr560, His920, and Cys917 36-37) or the molecular docking sites for UDP-GlcNAc (His498, Leu653, Tyr841, Gln839, Lys430, and His920) (Supplementary Figure 4E). Moreover, molecular dynamics simulations further confirm that after kaempferol binding, UDP-GlcNAc can still bind to OGT, forming a stable ternary complex (Supplementary Figure 4F). Notably, our enzyme activity assays provide direct evidence: in the wild-type OGT (WT) reaction system, the addition of kaempferol significantly reduced the UDP product generated by OGT, with this inhibitory effect being time-dependent. More importantly, when we mutated the His901 and Asp925 residues in OGT, the inhibitory effect of kaempferol was completely abolished, and the UDP production levels were not significantly different from the untreated control group. These results not only confirm the specificity of kaempferol' s inhibition of OGT activity but also directly prove the critical role of His901 and Asp925 residues in this inhibition process. Thus, kaempferol binds to a site on OGT distinct from the active site, altering the enzyme's conformation and rendering it catalytically inactive, even when the substrate is bound. This confirms a non-competitive mode of inhibition. In conclusion, our data support the mechanism where kaempferol binds to OGT → disrupts protein stability → promotes degradation → reduces overall OGT abundance → inhibits enzyme activity, and also secondarily modulates HBP metabolism and the availability of the OGT substrate UDP-GlcNAc. This cascade mechanism overcomes the limitations of current OGT inhibitors, which only target the UDP-GlcNAc binding site, providing a new strategy for the development of selective OGT modulators.

Hsp47 serves as a crucial target protein through which kaempferol exerts its anti-CRC effects by modulating O-GlcNAcylation, providing a scientific foundation for the development of novel anticancer strategies. Notably, although multiple proteins may contribute to O-GlcNAcylation alterations during CRC progression, Hsp47 plays an indispensable role in facilitating procollagen maturation and secretion, maintaining tumor extracellular matrix (ECM) integrity, and enhancing tissue structural stability. In particular, its O-GlcNAc modification appears to be critically involved in CRC pathogenesis. Our research revealed three key findings. First, Hsp47 undergoes significant O-GlcNAcylation. Secondly, kaempferol can effectively reduce the modification level of the key O-GlcNAcylation site Ser76 on Hsp47, inhibit its expression, and promote its retention within the endoplasmic reticulum (ER). Third, this reduced post-translational modification impairs Hsp47's ability to bind to type I pro-collagen molecules in the tumor ECM and transport them to the Golgi apparatus, leading to the accumulation of immature type I pro-collagen within the ER, which subsequently inhibits the biosynthesis of collagen I. These coordinated mechanisms ultimately lead to a significant inhibition of CRC growth.

In summary, we provide clear evidence that reducing the O-GlcNAcylation of the Hsp47 protein represents novel insight into the treatment of CRC. Although there have been no reports on the regulatory effects of glycosylation on collagen-related proteins during kaempferol-mediated inhibition of CRC, our findings significantly expand the understanding of Hsp47 and underscore the clinical potential of targeting Hsp47 O-GlcNAcylation as a therapeutic strategy for CRC.

## Conclusion

This study reveals that kaempferol suppresses colorectal cancer progression by dual targeting of glucose metabolism (reducing UDP-GlcNAc flux) and OGT activity, thereby diminishing O-GlcNAcylation of Hsp47. Mechanistically, hypo-O-GlcNAcylation of Hsp47 disrupts its chaperone function in collagen I maturation, ultimately impairing CRC cell proliferation and migration. Importantly, we validate O-GlcNAcylated Hsp47 as a biomarker and propose pharmacological blockade of this modification as a biomarker-guided therapeutic strategy against CRC.

## Supplementary Material

Supplementary figures.

## Figures and Tables

**Table 1:** Sequences of Q‒PCR Primers Used

Gene	Primer	Sequence
HK1	HK1-F	CGCAGCTCCTGGCCTATTAC
	HK1-R	GAGCCGCATGGCATAGAGAT
HK2	HK2-F	AAGCAGTCACTGATGTGGCA
	HK2-R	GGACAAGTACACGATCCGCT
GPI	GPI-F	CCACCAGCAGACACACATCA
	GPI-R	GGCTGCAGAGAGCTACCATC
PGM3	PGM3-F	GGCTCCGGACGTTTACGATT
	PGM3-R	GCAGACTCAGGGCAGATAGC
GFAT	GFAT-F	CTCCGGCATCATGTGTGGTA
	GFAT-R	TTTTGCAGGCATTGGCTTCC
UAP1	UAP1-F	AGACTGTGGAGCAAAGGTGG
	UAP1-R	ATTGAACAGCAGTCGTCCGT
OGT	OGT-F	GGGGTACCATGGCGTCTTCCGTGGGCAAC
	OGT-R	CGGGATCCTTATGCTGACTCAGTAC
OGA	OGA-F	CCGCTCGAGATGGTGCAGAAGGAGAGTC
	OGA-R	GGGGTACCTCACAGGCTCCGACCAAG
GAPDH	GAPDH-F	CCATGGGGAAGGTGAAGGTC
	GAPDH-R	GCGCCCAATACGACCAAATC

**Table 2:** Proteins with molecular weights between 20-50 kDa

SYMBOL	PXD012254	PXD019504	PXD014511	FC Average	Molecular mass(kDa)
NNMT	20.561	1.627	-	11.094	29
P5CR1	2.639	4.821	2.248	3.236	33
ANXA3	3.871	3.054	2.665	3.197	36
Hsp47	3.610	1.777	-	2.693	47
CNN2	-	2.315	2.799	2.557	33
FRIL	2.657	-	2.296	2.476	20
ARL8A	2.629	-	2.036	2.333	21
LACB2	-	2.609	2.045	2.327	30
PYR1	2.738	1.913	-	2.325	24
GAR1	-	2.328	1.800	2.064	22
ASSY	-	1.744	2.338	2.041	46
IPYR	2.245	1.956	1.774	1.992	32
OFUT1	2.283	1.621	-	1.952	43
RCN1	2.060	1.795	-	1.928	38
GPX2	1.620	-	2.074	1.847	21
RPAB1	1.732	-	1.684	1.708	24
VDAC1	1.863	-	1.549	1.706	30
RM04	1.724	1.617	-	1.670	34
PRKDC	1.595	1.730	-	1.663	46
LDHA	1.792	1.524	-	1.658	36
PA1B3	-	1.673	1.600	1.637	25
HCD2	1.615	1.650	-	1.632	26
PON2	-	1.564	1.685	1.624	39
CBX3	1.705	1.541	-	1.623	20
ITPR3	-	1.553	1.691	1.622	30
5MP1	1.735	1.508	-	1.622	48
FAS	1.554	-	1.663	1.608	27
CNOT1	1.535	1.655	-	1.595	26
PSME3	1.593	1.575	-	1.584	29
HACD3	1.542	1.620	-	1.581	43
UCHL3	-	1.649	1.501	1.575	26
CATH	-	1.517	1.554	1.536	37
IF2B	-	1.525	1.513	1.519	38

**Table 3:** Glycosylation site analysis

SYMBOL	O-GlcNAc-v2.0 (Score)	YinOYang-1.2 (Potential)	GPP	O-GlcNAc PRED-DL	O-GlcNAc Atlas
			(number)		
PRKDC	14	0.50-0.69(13)	253	182	9
FAS	24	0.50-0.67(24)	147	111	3
ITPR3	12	0.51-0.68(15)	156	102	3
PYR1	20	0.51-0.61(14)	135	97	7
CNOT1	21	0.50-0.66(21)	179	93	81
VDAC1	21	0.62(1)	25	23	1
Hsp47	14	0.50-0.58(3)	24	23	4
GPX2	-	0.43(1)	17	18	-
P5CR1	14	0.50-0.61(4)	21	17	4
LDHA	16	0.40-0.45(6)	17	16	2
RM04	11	0.52-0.58(4)	23	16	-
5MP1	6	0.52-0.57(3)	27	15	2
CNN2	12	0.50-0.62(2)	24	15	3
ANXA3	5	0.40-0.49(5)	23	15	-
OFUT1	7	0.58(1)	18	15	-
ASSY	19	0.53-0.61(4)	21	14	3
PSME3	12	0.51-0.64(2)	21	13	-
UCHL3	11	0.57-0.61(5)	12	12	-
HACD3	9	0.72-0.73(2)	19	12	-
HCD2	29	0.50-0.52(2)	10	12	1
CATH	-	0.40-0.56(11)	22	11	-
PON2	12	0.51-0.60(2)	23	10	1
NNMT	9	0.40-0.50(7)	17	10	-
ARL8A	17	0.40-0.45(3)	12	9	-
IPYR	13	0.42-0.45(3)	16	8	-
LACB2	9	0.49(1)	12	8	-
RCN1	21	0.31-0.32(4)	15	7	11
RPAB1	11	0.47-0.49(2)	11	6	-
CBX3	17	0.40-0.42(2)	12	6	-
IF2B	7	0.40-0.58(4)	22	5	-
FRIL	-	0.41-0.52(4)	7	5	-
PA1B3	7	0.59(1)	7	3	-
GAR1	11	0.30-0.39(4)	2	3	-

**Table 4:** Subcellular localization of the eight candidate proteins

SYMBOL	LOG RANK P	Subcellular Location
NNMT	0.013	Cytoplasm
Hsp47	0.042	Endoplasmic Reticulum (ER)
ITPR3	0.061	Endoplasmic Reticulum Membrane
CBX3	0.088	Nucleoplasm and Nucleus
ARL8A	0.089	Cytoplasm
LDHA	0.093	Cytoplasm
5MP1	0.290	Plasma Membrane and Cytoplasmic Matrix
RCN1	0.350	Nucleoplasm, Cytoplasm, and Nucleolus
