# Supplementary material for: O-GlcNAcylated Hsp47 as a predictive biomarker in colorectal cancer: Kaempferol targets OGT-collagen axis for therapeutic intervention
Source: Int J Biol Sci. 2025 Aug 30;21(12):5586–608. doi: 10.7150/ijbs.116513 (PMC12435574; doi:10.7150/ijbs.116513)
Supplement: Supplementary file 1 — Supplementary figures. [file ijbsv21p5586s1.pdf]

Supplemental Figure 1. Comprehensive antibody detection of 11 posttranslational modifications across 8 cell lines at various concentrations of kaempferol.

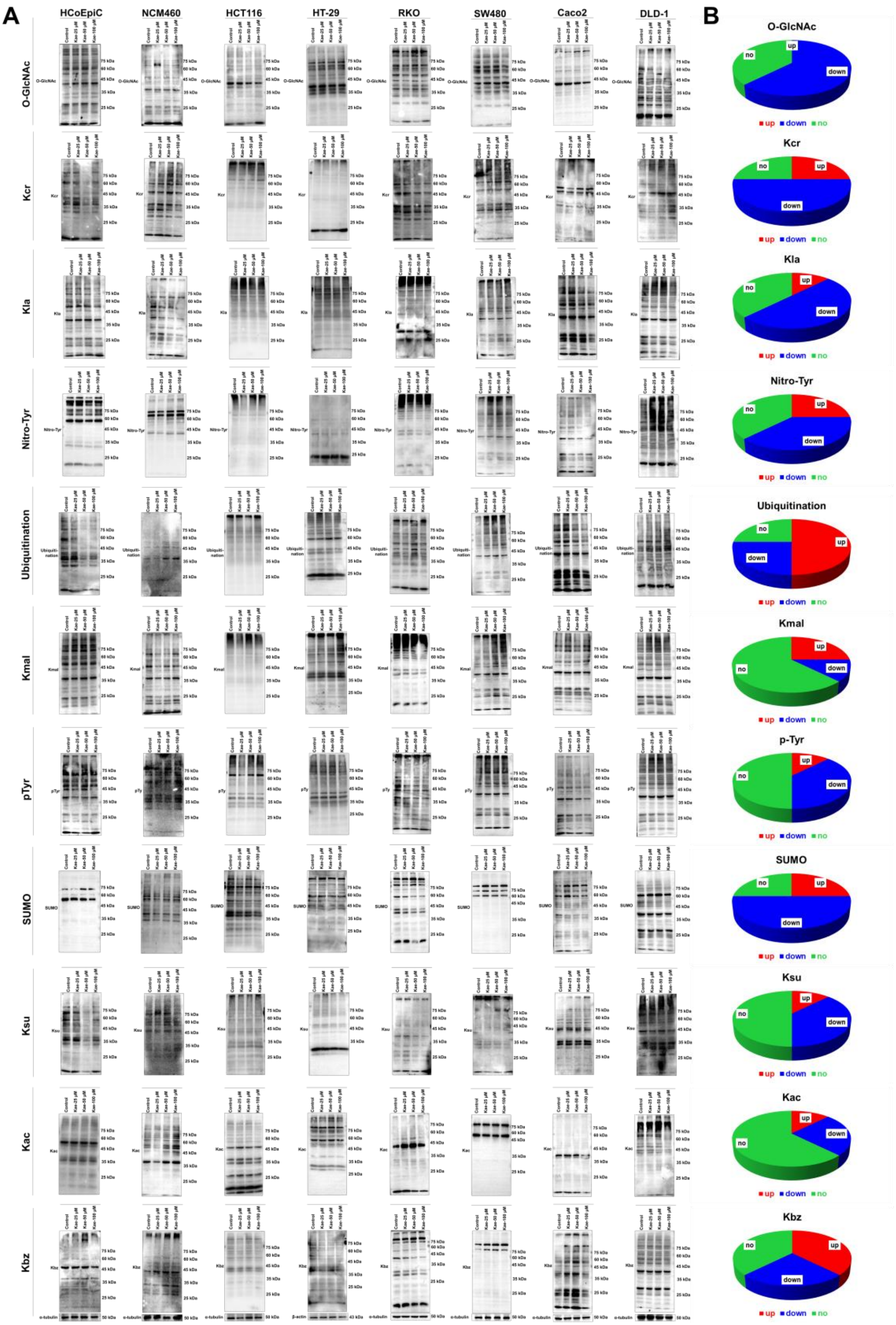

Supplementary Figure 2. Expression and role of OGT and OGA in CRC.

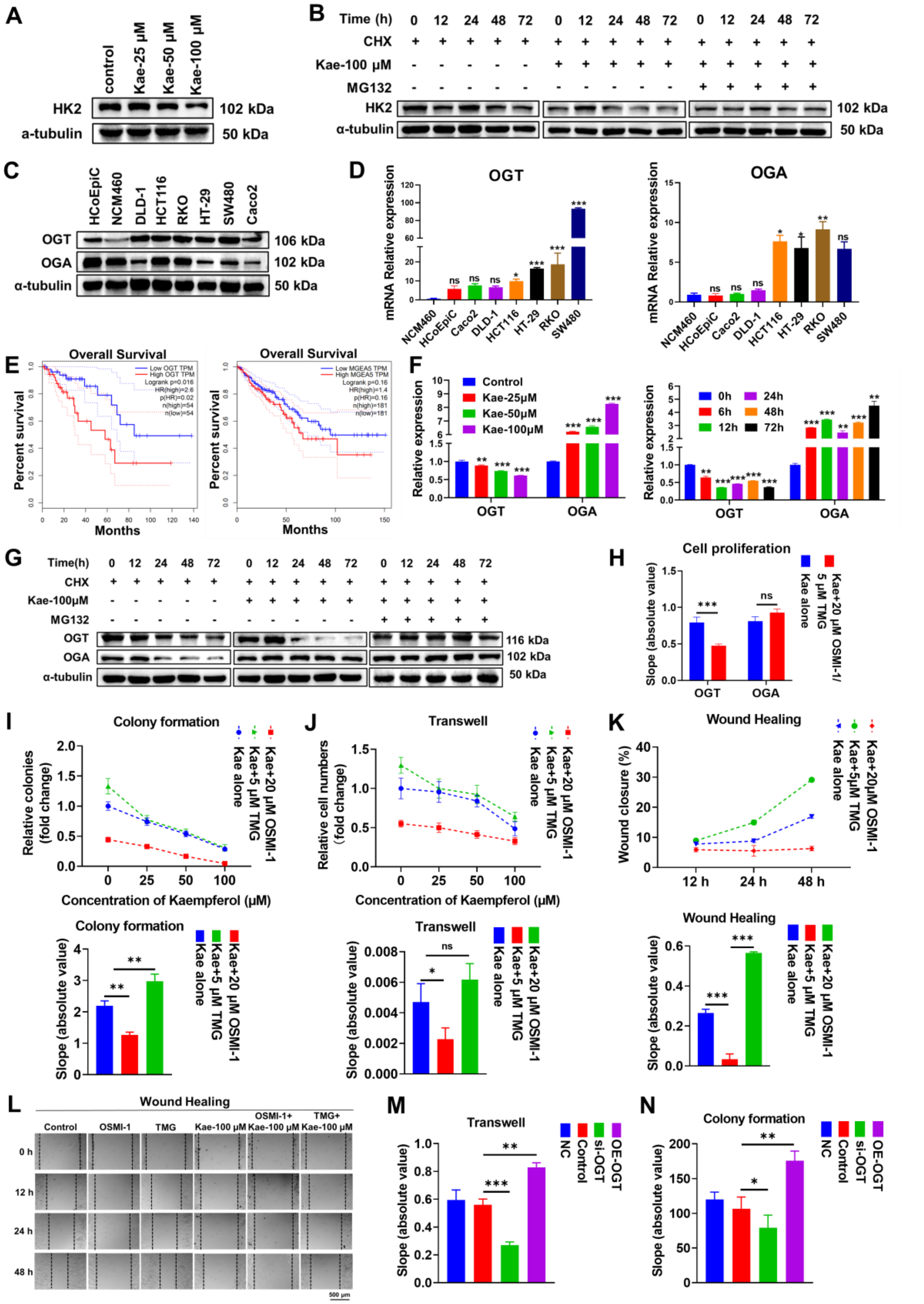

A, Effect of kaempferol on HK2 protein expression. **B**, Effect of kaempferol on HK2

protein stability. **C**, Differences in OGT and OGA protein expression between normal colonic epithelial cells and CRC cell lines. **D**, Differences in OGT and OGA mRNA expression between normal colonic epithelial cells and CRC cell lines. **E**, Survival analysis on the basis of OGT and OGA expression. **F**, Quantitative analysis of the effects of different concentrations of kaempferol on OGT and OGA protein expression at different time points. **G**, Effect of kaempferol on the half-life of OGT and OGA proteins. **H**, Quantitative analysis of the effects of OGT and OGA inhibition on the ability of kaempferol to suppress HCT116 cell viability. **I**, Quantitative analysis of the effects of OGT and OGA inhibition on the ability of kaempferol to suppress HCT116 cell stemness. **J**, Quantitative analysis of the effects of OGT and OGA inhibition on the ability of kaempferol to suppress HCT116 cell migration. **K**, Quantitative analysis of the effects of OGT and OGA inhibition on kaempferol-mediated suppression of HCT116 cell wound healing. **L**, Effects of OGT and OGA inhibition on HCT116 cell wound healing. **M**, Quantitative analysis of the effects of OGT knockdown or overexpression on the ability of kaempferol to suppress HCT116 cell migration. **N**, Quantitative analysis of the effects of OGT knockdown or overexpression on the ability of kaempferol to suppress HCT116 cell stemness. The data are presented as the means ± SDs, * $P < 0.05$, ** $P < 0.01$, *** $P < 0.001$, ns indicates no statistically significant difference.

Supplementary Figure 3. Binding analysis of kaempferol with OGA.

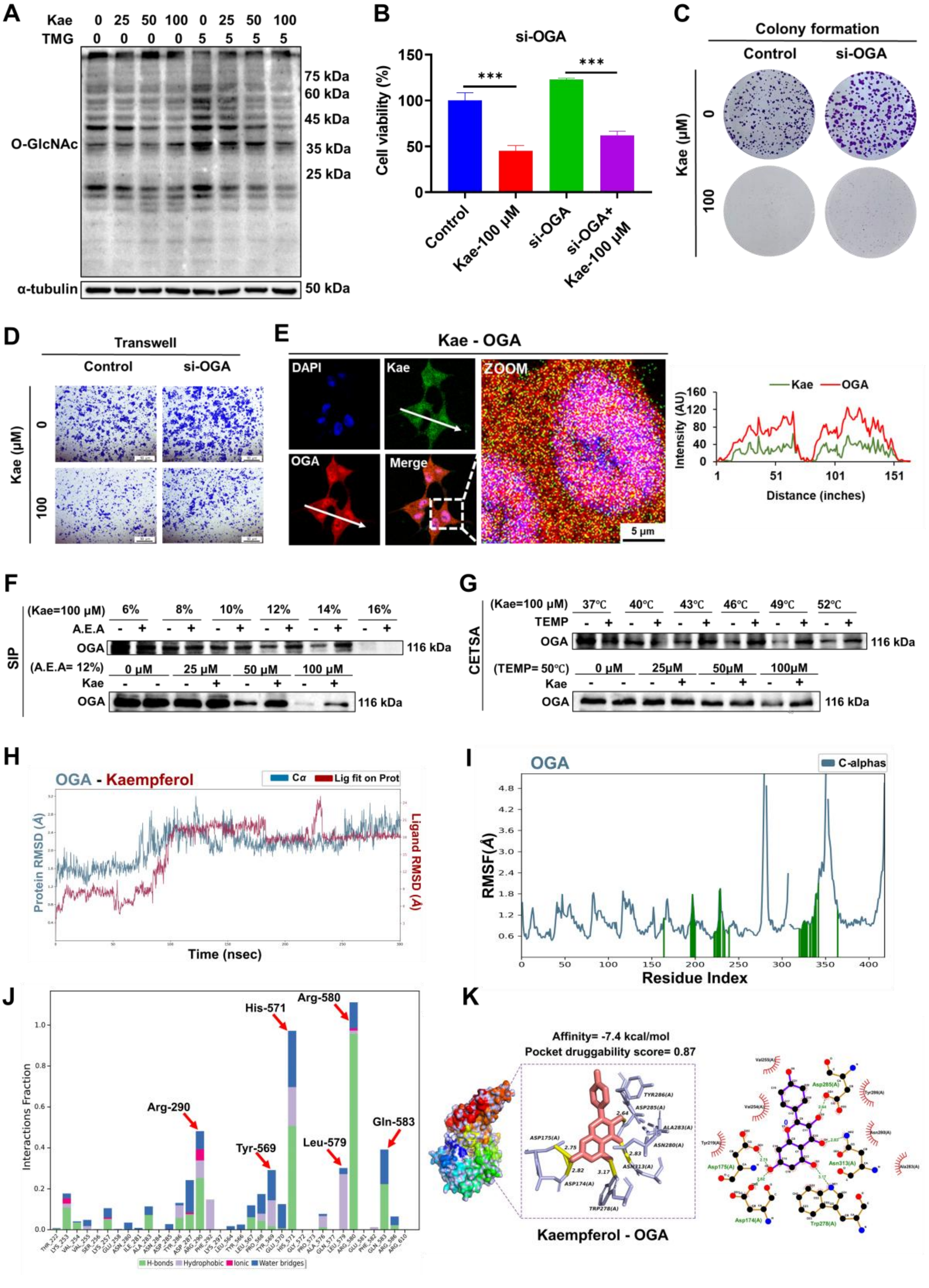

A, Western blotting analysis of the effect of combining kaempferol with the OGA inhibitor TMG on protein O-GlcNAcylation. **B**, The effect of OGA knockdown on the

inhibition of cell proliferation by kaempferol. **C**, The effect of OGA knockdown on the inhibition of cell stemness by kaempferol. **D**, The effect of OGA knockdown on the inhibition of cell migration by kaempferol. **E**, Fluorescence colocalization of kaempferol with OGA. **F**, SIP assay verifying the binding interaction between kaempferol and OGA. **G**, CETSA assay verifying the binding interaction between kaempferol and OGA. **H**, Root mean square deviation (RMSD) changes in OGA during molecular dynamics simulation with kaempferol. **I**, RMSF changes in the OGA during the molecular dynamics simulation. **J**, Changes in various interaction bonds between kaempferol and OGA during molecular dynamics simulation. **K,** Molecular docking analysis of kaempferol with OGA. The data are presented as the means ± SDs, * $P < 0.05$, ** $P < 0.01$, *** $P < 0.001$, ns indicates no statistically significant difference.

Supplementary Figure 4. Binding analysis of kaempferol/UDP-GlcNAc with OGT.

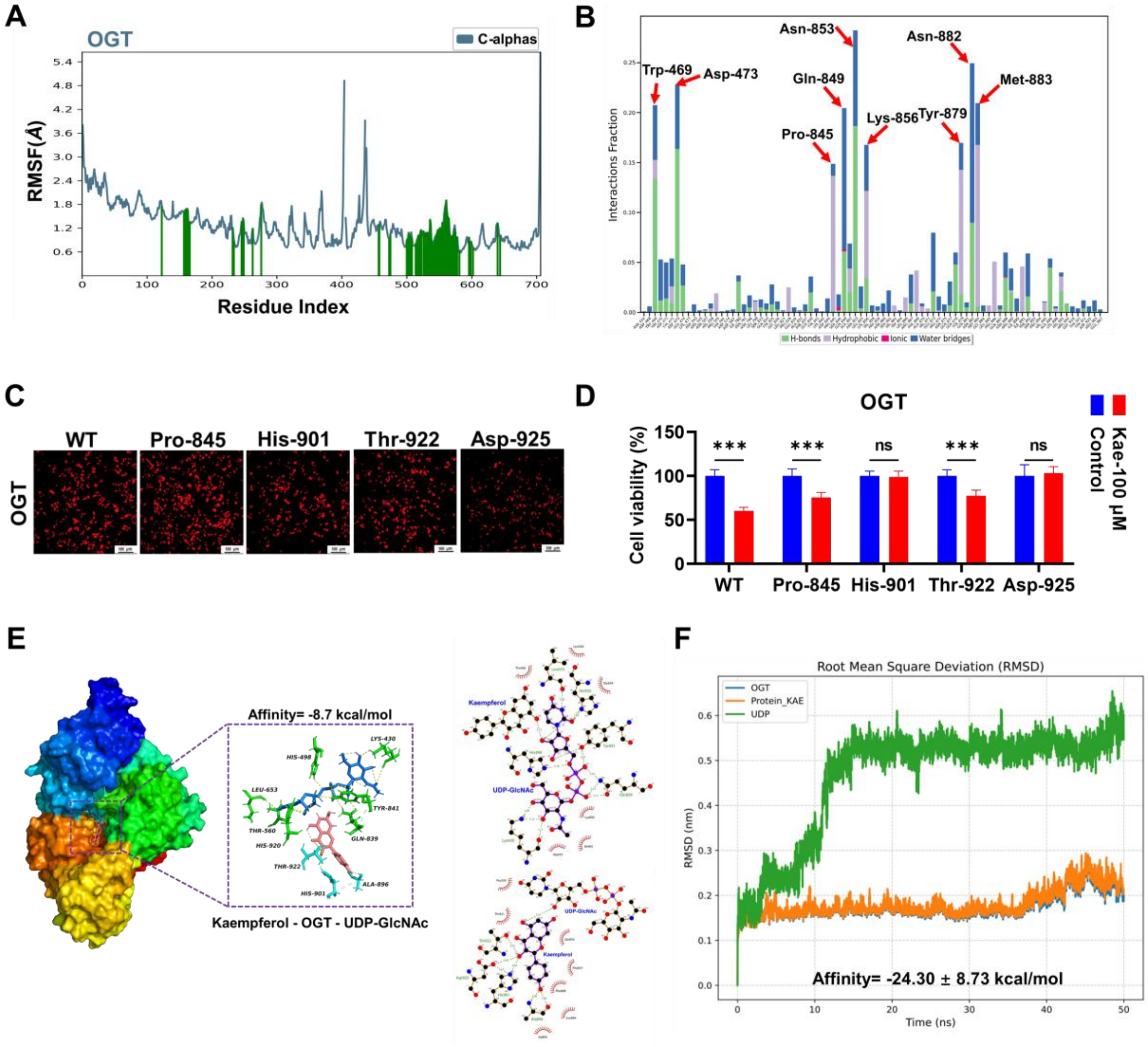

A, Root mean square fluctuation (RMSF) changes in OGT during molecular dynamics simulation. **B**, Analysis of interactions between OGT and kaempferol throughout the simulation, categorized into hydrogen bonds, hydrophobic interactions, ionic bonds, and water bridges. **C**, Fluorescence detection of OGT expression after key amino acid site mutation. **D**, Effects of mutations at the OGT binding sites of kaempferol on the inhibition of cell viability. **E,** Molecular docking of UDP-GlcNAc with kaempferol and OGT complex. **F,** Molecular dynamics simulation of UDP-GlcNAc with kaempferol and OGT complex. The data are presented as the means ± SDs, * $P < 0.05$, ** $P < 0.01$, *** $P < 0.001$, ns indicates no statistically significant difference.

Supplementary Figure 5. Analysis of the correlation between Hsp47 and colorectal cancer.

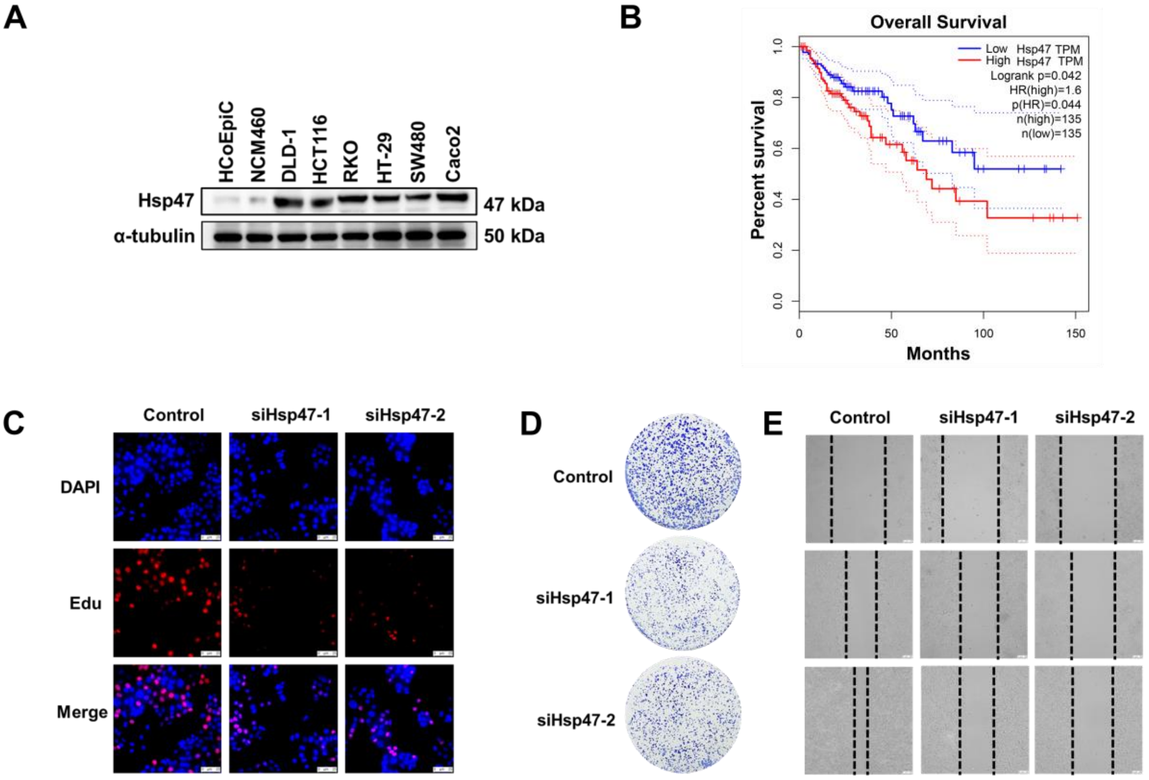

A, Protein expression of Hsp47 in normal intestinal epithelial cells and CRC cells. **B**, Survival analysis of Hsp47 in CRC patients. **C**, Edu detection of the inhibitory effect of Hsp47 knockdown on CRC cell proliferation. **D**, Plate colony formation assay to detect the inhibition of CRC cell stemness by Hsp47 knockdown. **E**, The cell scratch assay detects the inhibition of CRC cell migration by Hsp47 knockdown.

Supplementary Figure 6. The inhibitory effect of combining kaempferol with the chemotherapy drug oxaliplatin on CRC.

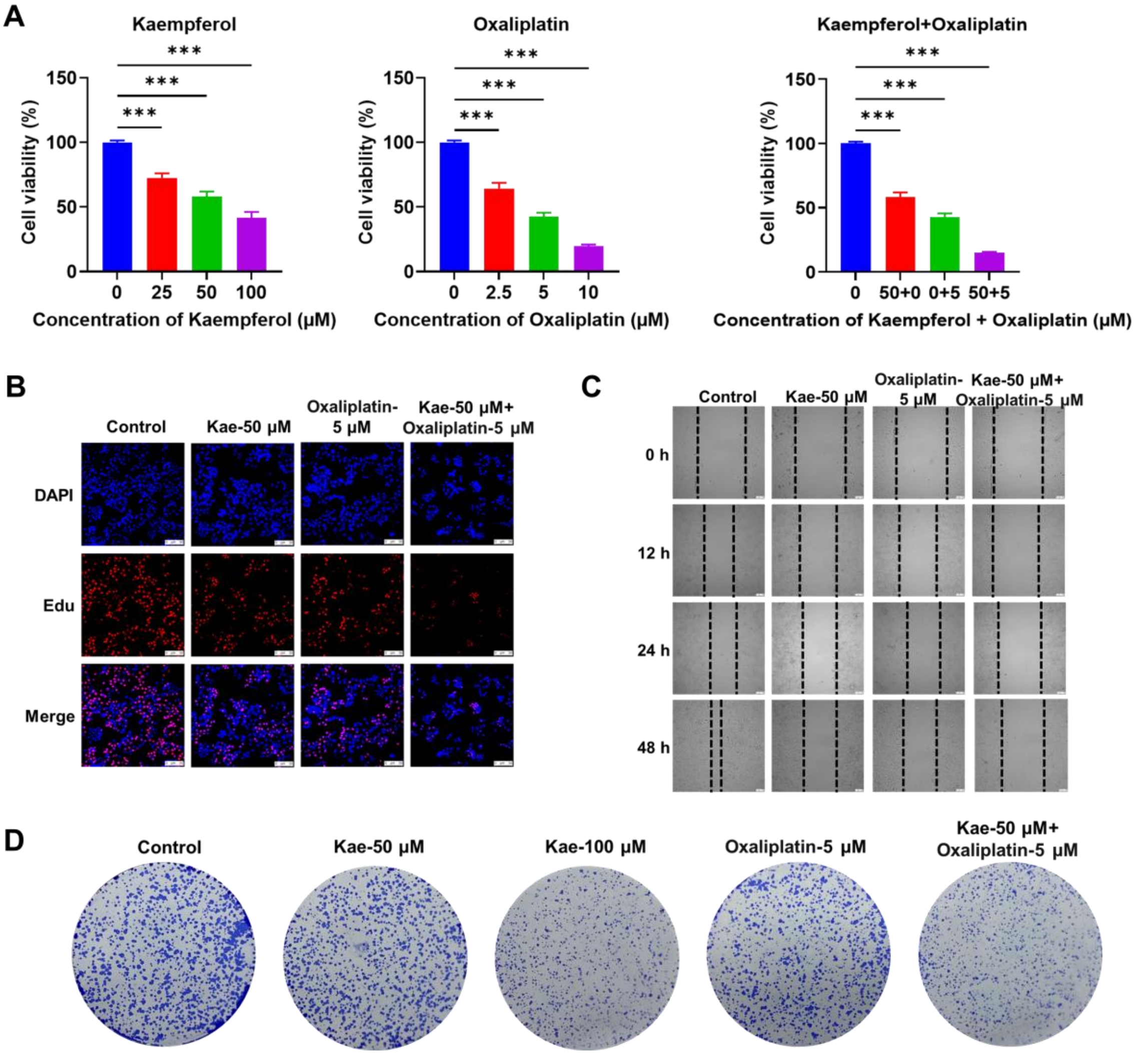

A, The SRB assay was used to evaluate the effect of kaempferol combined with oxaliplatin on CRC cell proliferation. **B**, Edu experiment to evaluate the effect of kaempferol combined with oxaliplatin on CRC cell proliferation. **C**, Wound healing assay to evaluate the effect of kaempferol combined with oxaliplatin on CRC cell migration. **D**, Colony formation assay to evaluate the effect of kaempferol combined with oxaliplatin on the stemness of CRC cells. The data are presented as the means ± SDs, * $P < 0.05$, ** $P < 0.01$, *** $P < 0.001$, ns indicates no statistically significant difference.
